# Human cytomegalovirus exploits host chromatin remodeler MORC2 to promote the DNA damage response on viral genomes

**DOI:** 10.1371/journal.ppat.1014693

**Published:** 2026-10-14

**Authors:** Jiseok Jeong, Dongjoon Jeong, Sungwon Lee, Junhyun Park, Hyewon Kim, Kwangseog Ahn

**Affiliations:** 1 Center for RNA Research, Institute for Basic Science, Seoul, Republic of Korea; 2 School of Biological Sciences, Seoul National University, Seoul, Republic of Korea; 3 The Research Institute of Basic Sciences, Seoul National University, Seoul, Republic of Korea; 4 SNU Institute for Virus Research, Seoul National University, Seoul, Republic of Korea; The Children’s Hospital of Philadelphia, UNITED STATES OF AMERICA

## Abstract

Human cytomegalovirus (HCMV) harbors a 230-kb double-stranded DNA genome whose replication induces extensive DNA damage during lytic infection. Because HCMV lacks canonical DNA repair machinery, it relies on host DNA damage response (DDR) pathways. However, the mechanisms that govern the preferential recruitment and function of host DDR proteins on viral genomes remain poorly understood. Here, we show that HCMV co-opts the host chromatin remodeler MORC Family CW-type zinc finger 2 (MORC2) to promote DDR activation on viral genomes. HCMV infection induces MORC2 expression, which is important for efficient viral DNA replication and progeny virus production. We found that MORC2 redistributes to viral replication compartments (vRCs) in HCMV-infected cells and enhances viral chromatin accessibility in an ATPase-dependent manner, thereby facilitating γH2AX accumulation and the recruitment of DNA repair factors to vRCs. Furthermore, we found that HCMV UL112-113-mediated phase separation facilitates MORC2 recruitment to vRCs, promoting efficient DDR signaling on viral genomes. Together, these findings provide a mechanistic framework for how DNA viruses alleviate replication stress by hijacking host chromatin remodelers.

## Author summary

DNA viruses depend on host DNA damage response (DDR) to preserve genome integrity during replication, but the mechanisms underlying preferential recruitment of DDR machinery to viral genomes are poorly understood. We show that human cytomegalovirus (HCMV) co-opts the host chromatin remodeler MORC2 to promote viral chromatin accessibility, DDR activation, and subsequent repair factor accumulation on viral genomes. UL112–113-mediated phase separation promotes MORC2 recruitment into replication compartments, thereby facilitating DDR activation on viral genomes. These findings reveal a mechanism by which a DNA virus hijacks host genome maintenance functions on its own DNA and identify MORC2 as a key host factor supporting HCMV replication.

## Introduction

Human cytomegalovirus (HCMV) belongs to the *Betaherpesvirinae* subfamily and is a ubiquitous pathogen with a seroprevalence of 40–90% [1,2]. In immunocompromised individuals, HCMV can cause severe diseases such as pneumonitis, hepatitis, retinitis, and gastrointestinal disorders [3–6]. HCMV possesses an approximately 230-kb double-stranded DNA genome, one of the largest among DNA viruses, and encodes nearly 200 open reading frames (ORFs) [7,8]. For efficient lytic replication, HCMV manipulates host cell cycle progression to create a favorable environment for viral replication. Several viral proteins, including UL69 and IE1, inhibit the G1/S transition [9–11], thereby preventing host DNA synthesis and reducing competition for cellular replication resources. While host DNA replication is suppressed during infection, HCMV initiates robust viral DNA replication within viral replication compartments (vRCs), which are distinct subnuclear structures specialized to viral DNA replication [12]. A recent study has shown that vRCs are formed through liquid-liquid phase separation (LLPS), and that these biomolecular condensates facilitate the recruitment of viral factors such as UL44, the viral polymerase processivity factor [13]. Given that LLPS is a protein interaction hub [14–16], it remains to be elucidated which host and viral proteins are recruited to vRCs and how they contribute to promoting viral replication.

During viral DNA replication, HCMV modulates the host DNA damage response (DDR) to support efficient replication during infection [17–20]. It has been reported that the DNA damage occurs depending on HCMV DNA replication, and repair of this damage is crucial for efficient lytic infection [19,21]. Consistent with this, several DDR marker proteins, including phosphorylated H2AX (γH2AX) and p53, mainly accumulate on viral genomes, despite some chromosomal breaks induced on the host genome during viral replication [18,21,22]. Furthermore, another study has demonstrated that the nucleotide excision repair proteins also localize to vRCs, engaging DNA repair on viral DNA [19]. Despite the preferential accumulation of DDR factors on viral genomes, it remains unclear how these factors are selectively recruited to vRCs over the damaged host chromatin and which host or viral factors establish this preferential DDR engagement on viral genomes.

The DDR pathway proceeds sequentially through three major steps: damage sensing, signal transduction, and effector recruitment [23,24]. Chromatin remodelers play an essential role in initiating this process by promoting nucleosome eviction and repositioning at sites of damage, which increases the accessibility for DDR factors among histone-packed DNA [25–27]. HCMV DNA rapidly undergoes chromatinization immediately after nuclear entry [28] and genome-wide nucleosome profiling has further revealed that nucleosome occupancy at different loci of the viral DNA changes dynamically throughout lytic infection. Such dynamic nucleosome profiles, coupled with robust DDR signaling during viral DNA replication, suggest that chromatin remodeling is highly active throughout infection [29]. However, the chromatin remodelers responsible for modulating the viral nucleosome landscape and facilitating efficient DDR activation on the chromatinized viral DNA remain largely unidentified.

MORC Family CW-Type Zinc Finger 2 (MORC2), one of the chromatin remodelers, has been recently identified as a critical regulator that facilitates the DDR through its ATPase-dependent chromatin remodeling activity [30,31]. MORC2 can regulate DDR; for instance, it induces γH2AX through its ATPase activity and promotes the subsequent recruitment of repair proteins [32]. Although MORC2 dysfunction has been associated with various pathological conditions, such as Charcot-Marie-Tooth disease, breast cancer, and hepatocellular carcinoma [33–36], there have been no reports on MORC2-mediated DDR in the context of host-pathogen infection.

## Results

### HCMV engages MORC2 to promote productive lytic replication

To characterize the function of MORC2 during HCMV lytic infection, we first examined the kinetics of MORC2 expression in infected cells. Wild-type (WT) HCMV (Toledo strain) infection induced MORC2 expression, which began to increase at 24 hours post-infection (hpi) and reached a peak at 48 hpi, showing a 2.9-fold increase relative to the mock-infected control. In contrast, infection with UV-inactivated HCMV failed to induce MORC2 expression, indicating that MORC2 upregulation requires active viral gene expression and/or downstream replication-associated events (Fig 1A). Additionally, we examined MORC2 expression kinetics using the TB40/E strain. Consistent with the results obtained with the Toledo strain, MORC2 expression was induced by WT TB40/E but not by UV-inactivated TB40/E (S1A Fig). We next assessed the subcellular localization of endogenous MORC2 during HCMV infection. In mock-infected cells, MORC2 displayed a diffuse pan-nuclear distribution. By 24 hpi, MORC2 became enriched in discrete nuclear foci that overlapped with UL44-positive pre-vRCs. This enrichment was maintained as the vRCs expanded and matured through 48 and 72 hpi (Fig 1B). These observations indicate that vRCs are a prominent site of MORC2 accumulation during HCMV infection.

**Fig 1:**
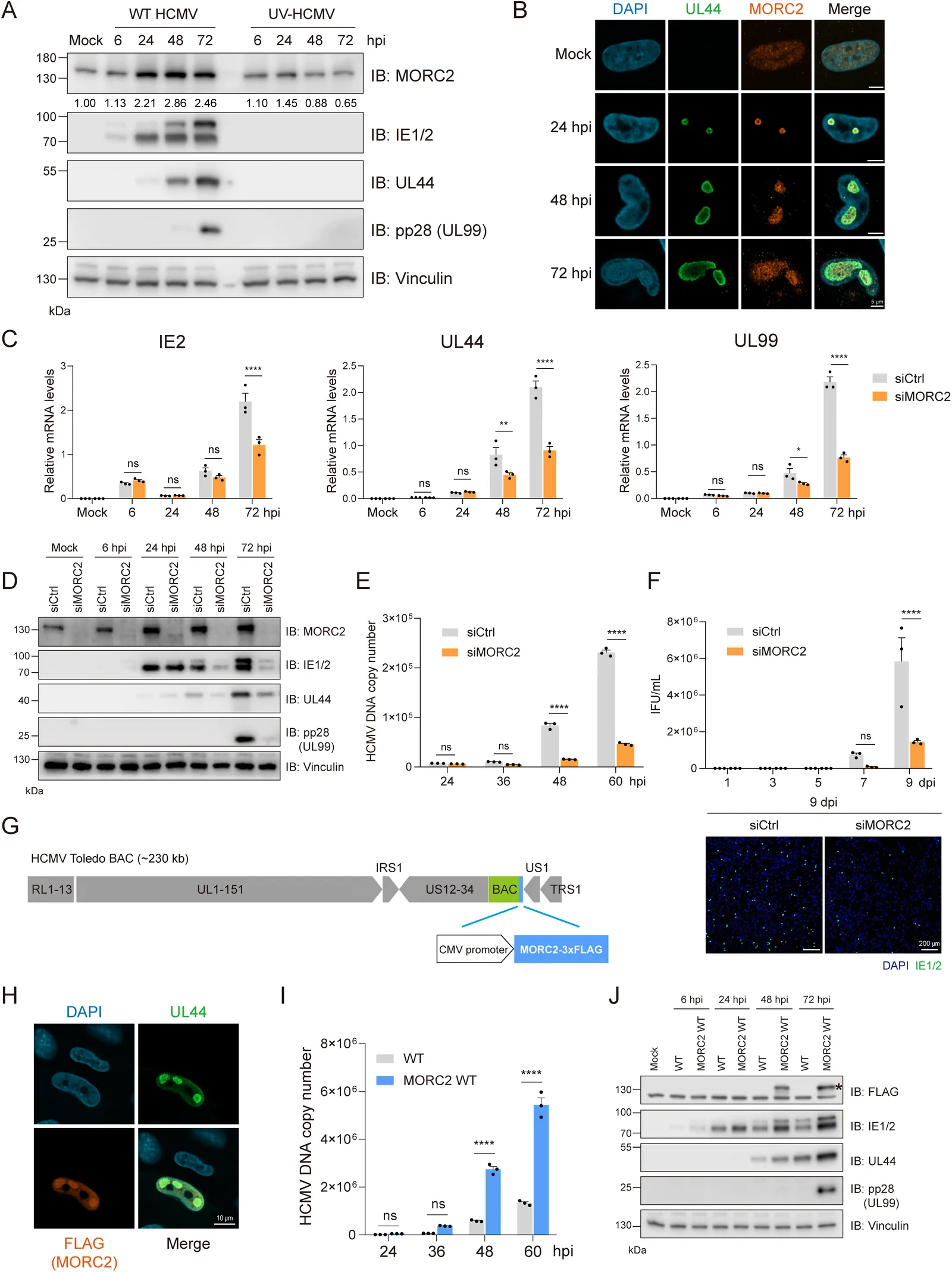
HCMV utilizes MORC2 to support efficient lytic replication. **(A)** Western blot analysis of MORC2 and viral proteins (IE1/2, UL44, pp28) in HFFs infected with WT or UV-inactivated HCMV (MOI = 2) at the indicated hours post-infection (hpi). MORC2 levels were normalized to vinculin and are shown relative to those in mock-infected cells. Vinculin served as a loading control (n = 2). **(B)** Immunofluorescence analysis of HFFs infected with WT HCMV (MOI = 1) and fixed at the indicated time points. UL44 (green), MORC2 (orange), and DAPI (light blue) are shown. Scale bar, 5 μm. (C) qRT-PCR analysis of viral transcripts (IE2, UL44, UL99) in siCtrl- or siMORC2-transfected HFFs infected with HCMV (MOI = 1). Cells were harvested at the indicated time points (mean ± SEM, n = 3). Two-way ANOVA with Sidak’s multiple comparisons test. **(D)** Immunoblot analysis of MORC2 and viral proteins (IE1/2, UL44, pp28) in siCtrl- or siMORC2-transfected HFFs infected with HCMV (MOI = 1). Cells were harvested at the indicated time points. Vinculin served as a loading control (n = 2). **(E)** Viral DNA copy number quantified by qPCR in siCtrl- or siMORC2-transfected HFFs infected with HCMV (MOI = 1). Cells were harvested at the indicated time points (mean ± SEM, n = 3). Two-way ANOVA with Sidak’s multiple comparisons test. **(F)** Top, cell-free progeny virus in the supernatant was quantified by limiting dilution analysis at the indicated time points from siCtrl- or siMORC2-transfected HFFs infected with HCMV (MOI = 0.1) (mean ± SEM, n = 3). Bottom, representative immunofluorescence images of fresh HFFs infected with 100-fold diluted cell-free supernatants collected at 9 dpi from siCtrl- or siMORC2-transfected HFFs. Cells were stained for IE1/2 (green) and counterstained with DAPI (blue). Two-way ANOVA with Sidak’s multiple comparisons test was performed across the time course; statistical comparisons at 7 and 9 dpi are shown. Scale bar, 200 μm. **(G)** Schematic representation of recombinant MORC2 WT HCMV BAC. The MORC2-3xFLAG cassette was inserted into the US1-US2 intergenic region under the control of the CMV promoter in the Toledo HCMV BAC (~230 kb). **(H)** Immunofluorescence analysis of HFFs infected with MORC2 WT HCMV (MOI = 1) and fixed at 48 hpi. UL44 (green), FLAG (orange), and DAPI (light blue) are shown. Scale bar, 10 μm. **(I)** Viral DNA copy number quantified by qPCR in HFFs infected with WT or MORC2 WT HCMV (MOI = 1) at the indicated time points (mean ± SEM, n = 3). Two-way ANOVA with Sidak’s multiple comparisons test. **(J)** Immunoblot analysis of FLAG and viral proteins (IE1/2, UL44, pp28) in HFFs infected with WT or MORC2 WT HCMV (MOI = 1). Cells were harvested at the indicated time points. A black asterisk indicates MORC2-3xFLAG. Vinculin served as a loading control (n = 2).

To investigate the role of MORC2 during HCMV infection, we depleted MORC2 using a pool of small interfering RNAs (siRNAs) prior to infection. We confirmed that mRNA levels of MORC2 were efficiently downregulated upon two days of siRNA treatment (S1B Fig). Intriguingly, MORC2 depletion in infected cells resulted in significantly reduced viral mRNA and protein levels. Notably, viral mRNA and protein levels remained comparable up to 24 hpi, prior to the onset of major viral DNA replication [37]. However, these defects became evident after 48 hpi, coinciding with robust viral DNA replication (48–72 hpi) [38] (Fig 1C and 1D). Likewise, viral DNA copy number was severely reduced in siMORC2-treated cells after 48 hpi (Fig 1E), which led to a significant reduction in infectious progeny production (Fig 1F). To rule out potential off-target effects, we repeated the experiments using individual siRNAs and observed a reduction in viral protein expression only when MORC2 was efficiently depleted (S1C Fig). Consistent with the results obtained using the Toledo strain, MORC2 depletion also reduced viral protein expression and infectious progeny production following infection with the TB40/E strain (S1D and S1E Fig). Furthermore, MORC2 depletion resulted in reduced viral protein expression in other HCMV-permissive cell types, including MRC-5 fibroblasts and U373MG glioblastoma cells, supporting a conserved pro-viral role for MORC2 across diverse cell types (S2A and S2B Fig). Collectively, these data indicate that HCMV utilizes MORC2 to support efficient viral DNA replication and subsequent gene expression during lytic infection.

To determine whether MORC2 contributes to HCMV DNA replication during lytic infection, we engineered a recombinant HCMV expressing 3xFLAG-tagged MORC2 (referred to as MORC2 WT HCMV). The CMV promoter-driven MORC2 expression construct was inserted into the US1-US2 intergenic region, which is not crucial for lytic infection [39,40] (Fig 1G and S1F Fig). This system allowed for the specific overexpression of MORC2 in HCMV-infected cells, and we had previously established this expression strategy as a useful platform for investigating HCMV lytic infection without critically impairing infection progression [21]. Using the recombinant virus, we first assessed the localization of overexpressed MORC2. MORC2-3xFLAG was detected specifically in UL44-positive infected cells, but not in adjacent uninfected cells, confirming that the recombinant virus enabled infection-restricted MORC2 overexpression (Fig 1H). Similar to endogenous MORC2, MORC2-3xFLAG was enriched in UL44-positive vRCs at 24, 48, and 72 hpi (S1G Fig).

We next assessed how MORC2 overexpression affects HCMV lytic replication. Compared to WT HCMV, MORC2 WT HCMV exhibited similar viral DNA levels up to 36 hpi. However, after 48 hpi when viral DNA replication is active [38], viral DNA copy numbers were also significantly increased in MORC2 WT HCMV-infected cells (Fig 1I). Similarly, viral protein levels were elevated in the MORC2 WT HCMV-infected cells compared to the WT HCMV-infected cells after 48 hpi (Fig 1J), indicating the pro-viral function of MORC2 during HCMV lytic infection.

### HCMV leverages MORC2 to promote DDR signaling on viral genomes

Building on our findings that MORC2 accumulates at vRCs and supports efficient viral DNA synthesis, we next asked whether HCMV utilizes MORC2 to maintain viral genome integrity during replication. Because MORC2 functions as a chromatin remodeler and can facilitate DDR signaling [30,32], we investigated whether MORC2 promotes DDR activation on viral DNA during HCMV infection (Fig 2A). To test the role of MORC2 in DDR activation on the HCMV genome, we first examined the induction of γH2AX, a hallmark of DDR activation, by immunofluorescence assay. Consistent with previous reports [18,21], γH2AX was preferentially enriched within vRCs in control infected cells (Fig 2B). Interestingly, we found that, upon MORC2 depletion, γH2AX induction at vRCs was significantly reduced (Fig 2B).

**Fig 2:**
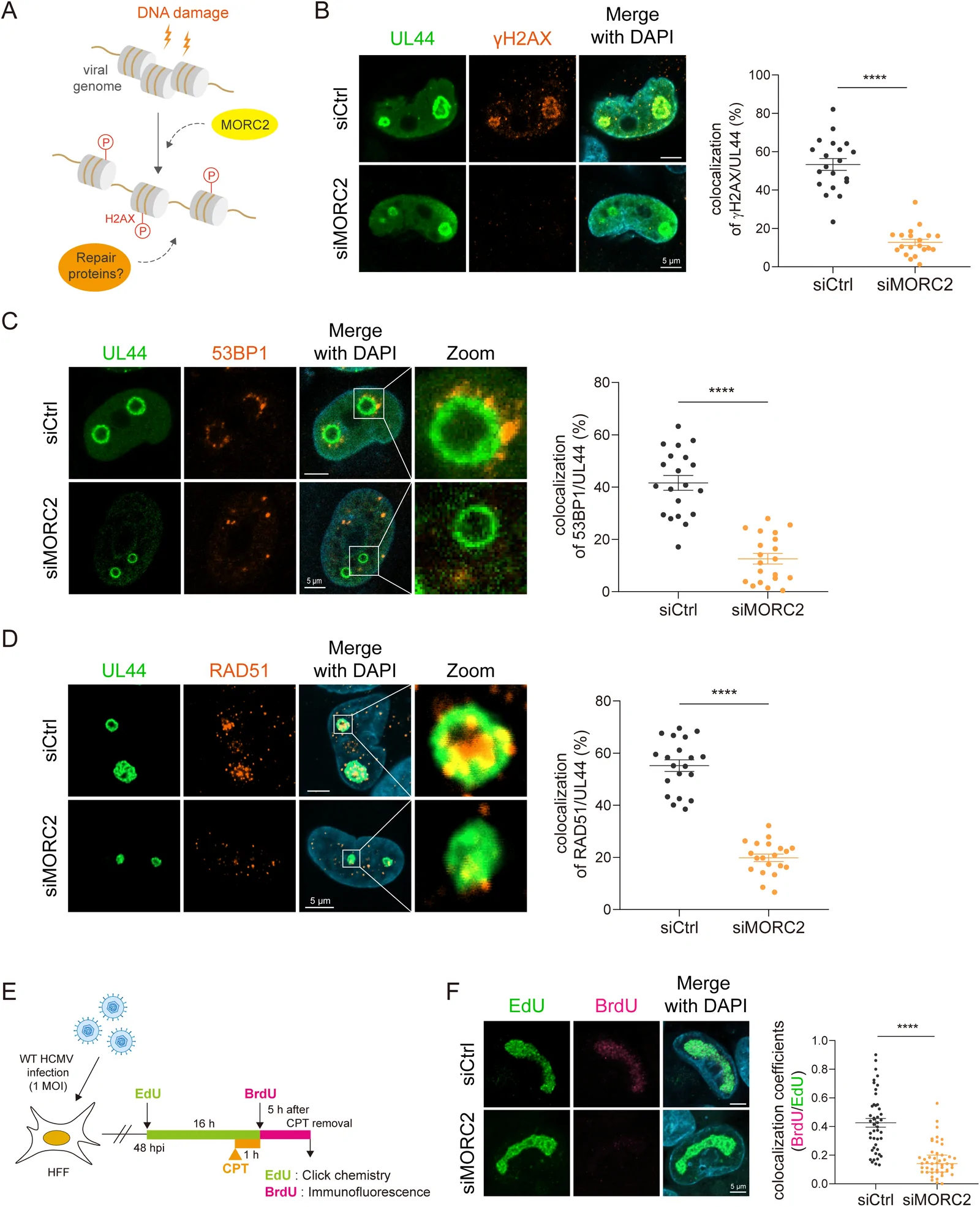
HCMV harnesses MORC2 for γH2AX signaling on viral genomes. **(A)** Model illustrating MORC2-dependent chromatin remodeling and DDR signaling (γH2AX) at vRCs, leading to repair factor accumulation on viral genomes. **(B)** Representative confocal images showing γH2AX (orange) enrichment within UL44-positive vRCs (green) in HFFs transfected with siCtrl or siMORC2 and subsequently infected with HCMV (MOI = 1). Cells were fixed at 48 hpi, and nuclei were counterstained with DAPI (light blue). Quantification shows the fraction of γH2AX signal overlapping with UL44-positive regions (mean ± SEM, n = 20 cells per condition from three independent experiments). Mann-Whitney test. **(C and D)** Representative confocal images showing enrichment of 53BP1 or RAD51 (orange) at UL44-positive vRCs (green) in HFFs transfected with siCtrl or siMORC2 and subsequently infected with HCMV (MOI = 1). Cells were fixed at 48 hpi, and nuclei were counterstained with DAPI (light blue). Quantification shows the fraction of 53BP1 or RAD51 signal overlapping with UL44-positive regions (mean ± SEM, n = 20 cells per condition from three independent experiments). Mann-Whitney test. **(E)** Schematic of the experimental design for assessing replication restart following CPT-induced replication stress. HFFs were infected with HCMV (MOI = 1) and labeled with EdU for 16 h starting at 48 hpi, with CPT added during the final 1 h of EdU labeling to induce replication stress. After CPT removal, cells were incubated for 5 h in the presence of BrdU to monitor resumption of DNA synthesis. EdU and BrdU were detected by click chemistry and immunofluorescence, respectively. **(F)** Quantification of the BrdU/EdU colocalization coefficient to assess replication restart following CPT-induced replication stress. The colocalization coefficient between BrdU (magenta) and EdU (green) signals was measured in HFFs transfected with siCtrl or siMORC2 and subsequently infected with HCMV (MOI =  1). Nuclei were counterstained with DAPI (light blue). Data are shown for individual cells (mean ± SEM, siCtrl, n = 45; siMORC2, n = 47, from three independent experiments). Unpaired t-test with Welch’s correction. Scale bars, 5 µm.

Intrigued by the strong effect of the MORC2 knockdown on γH2AX induction, we also tested if the DNA damage repair protein recruitment, which is downstream of DDR signaling, was also impaired upon MORC2 depletion. In control cells, 53BP1 and RAD51, which are central to the non-homologous end joining (NHEJ) and homologous recombination (HR) pathways, respectively [41,42], were both robustly recruited to vRCs (Fig 2C and 2D). In contrast, in MORC2-depleted cells, the localization of both 53BP1 and RAD51 to vRCs was substantially reduced compared to control cells (Fig 2C and 2D). Collectively, these results indicate that HCMV utilizes MORC2 to promote DDR signaling at vRCs, including γH2AX accumulation and the subsequent recruitment of repair factors.

To assess whether impaired DDR signaling contributes to the viral defects observed upon MORC2 depletion, we depleted 53BP1 and RAD51 individually or in combination. We confirmed efficient knockdown of RAD51 and partial depletion of 53BP1 (S3A Fig). Consistent with the effects of MORC2 depletion, knockdown of 53BP1 and/or RAD51 also resulted in reduced viral protein expression (IE1/2, UL44, and pp28) (S3B Fig) and impaired viral DNA replication (S3C Fig).

Since DNA repair proteins like 53BP1 and RAD51 are crucial for alleviating replication stress [43,44], we sought to determine the functional consequences of impaired MORC2-mediated DDR signaling. To this end, we performed an EdU/BrdU dual-labeling assay using camptothecin (CPT), a topoisomerase I inhibitor, to induce replication stress [45,46]. HCMV-infected cells were labeled with EdU from 48 to 64 hpi to mark actively replicating viral DNA, with CPT added during the final 1 h of EdU labeling to induce replication fork stalling. After CPT removal, cells were subsequently pulsed with BrdU for 5 h, which was detected separately from EdU, to monitor the recovery rate of DNA synthesis after chemically induced replication stress (Fig 2E). While control cells efficiently incorporated BrdU indicating successful alleviation of replication stress, MORC2-depleted cells showed markedly reduced resumption of viral DNA synthesis following CPT removal (Fig 2F). Furthermore, depletion of either 53BP1 or RAD51 phenocopied the defects seen with MORC2-depleted cells in the EdU/BrdU dual-labeling assay, as evidenced by a significant reduction in BrdU incorporation after CPT-induced replication stress (S3D Fig). Collectively, these findings suggest that HCMV exploits MORC2 to facilitate DNA repair factor accumulation at vRCs and support the resumption of viral DNA synthesis after replication stress.

### HCMV exploits MORC2 ATPase activity to enhance viral chromatin accessibility

To investigate the detailed molecular mechanism of MORC2-mediated DDR signaling, we examined whether MORC2 facilitates viral chromatin accessibility during HCMV DNA replication. Using a nuclease-based chromatin accessibility assay, we assessed the openness of viral chromatin by quantifying viral DNA via qPCR. We tested multiple regions of the HCMV genome (UL44, UL57, oriLyt, UL79, UL84, UL87, UL99, UL122, MIEP, UL138, US13, and US28) and included two host loci as controls, the NANOG promoter, a transcriptionally silenced region in differentiated somatic cells, and the promoter of ACTB, a constitutively active housekeeping gene (Fig 3A) [47,48]. Viral chromatin accessibility was significantly reduced across all tested viral loci upon MORC2 depletion. Notably, we did not detect any significant change in chromatin accessibility at the two control host loci, which is consistent with preferential enrichment of MORC2 to vRCs (Fig 3B).

**Fig 3:**
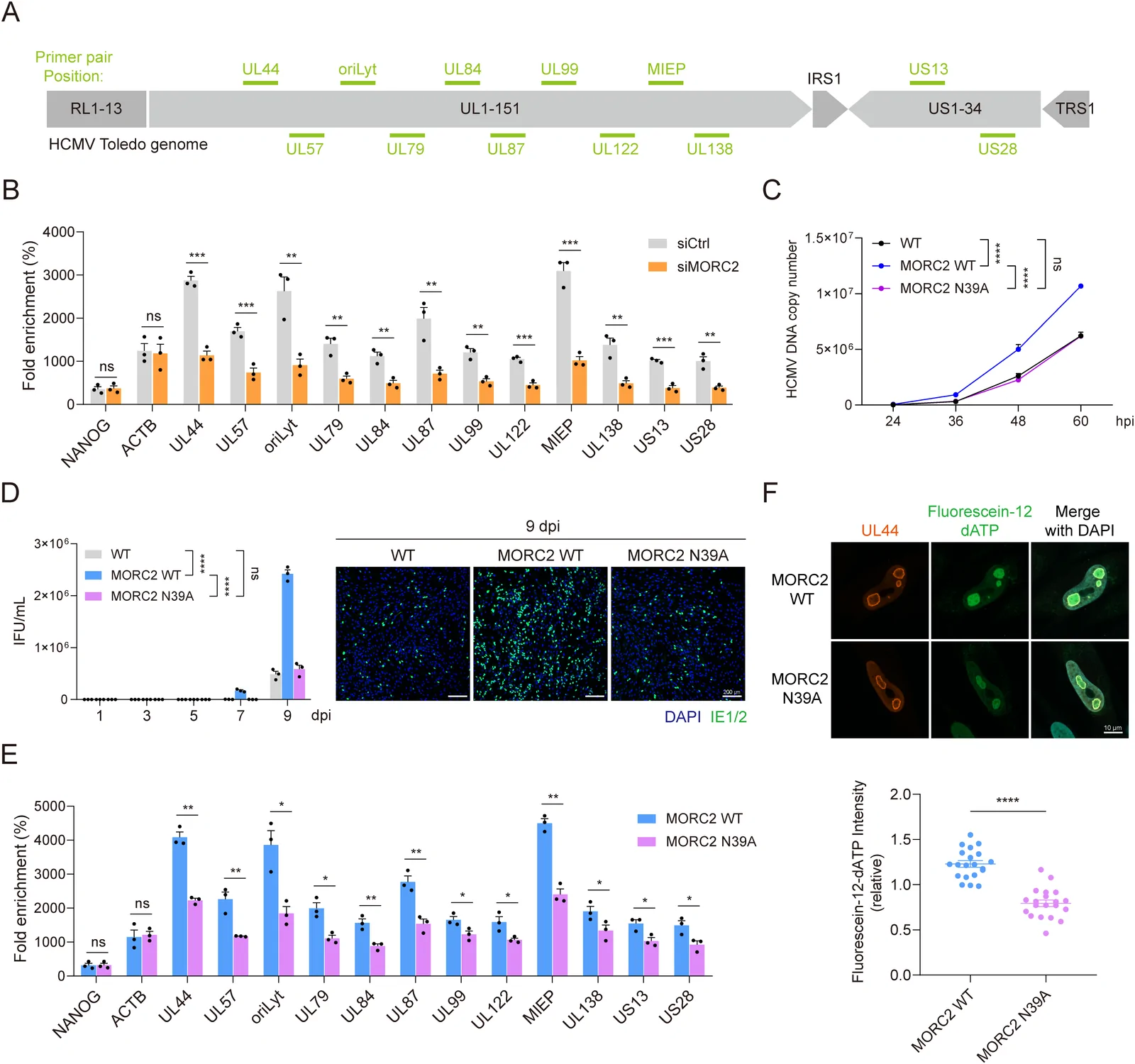
ATPase-dependent chromatin remodeling by MORC2 enhances viral chromatin accessibility. **(A)** Schematic representation of the HCMV Toledo genome and the locations of primer pairs used for chromatin accessibility assays. **(B)** Chromatin accessibility assay followed by qPCR at the indicated loci (NANOG, ACTB, UL44, UL57, oriLyt, UL79, UL84, UL87, UL99, UL122, MIEP, UL138, US13, US28) in siCtrl- and siMORC2-transfected cells during HCMV infection (MOI = 1). Data are presented as fold enrichment (%) (mean ± SEM, n = 3). Multiple unpaired t-tests with FDR correction (Benjamini, Krieger and Yekutieli method, Q = 5%). **(C)** Quantification of HCMV DNA copy number by qPCR in HFFs infected with WT, MORC2 WT or MORC2 N39A HCMV (MOI = 1). Cells were harvested at the indicated time points (mean ± SEM, n = 3). Two-way ANOVA with Sidak’s multiple comparisons test was performed across the time course; statistical comparisons at 60 hpi are shown. **(D)** Left, cell-free progeny virus in the supernatant was quantified by limiting dilution analysis at the indicated time points from HFFs infected with WT, MORC2 WT, or MORC2 N39A HCMV (MOI = 0.1) (mean ± SEM, n = 3). Right, representative immunofluorescence images of fresh HFFs infected with 10-fold diluted cell-free supernatants collected at 9 dpi. Cells were stained for IE1/2 (green) and counterstained with DAPI (blue). Two-way ANOVA with Sidak’s multiple comparisons test was performed across the time course; statistical comparisons at 9 dpi are shown. Scale bar, 200 μm. **(E)** Chromatin accessibility assay followed by qPCR at the indicated loci (NANOG, ACTB, UL44, UL57, oriLyt, UL79, UL84, UL87, UL99, UL122, MIEP, UL138, US13, US28) in HFFs infected with MORC2 WT or MORC2 N39A HCMV (MOI = 1). Data are presented as fold enrichment (%) (mean ± SEM, n = 3). Multiple unpaired t-tests with FDR correction (Benjamini, Krieger and Yekutieli method, Q = 5%). **(F)** Top, representative images from the NicE-view labeling assay using fluorescein-12-dATP in HFFs infected with MORC2 WT or MORC2 N39A HCMV (MOI = 1). Cells were immunostained for UL44 (orange), and nuclei were counterstained with DAPI (light blue). Bottom, quantification of fluorescein-12-dATP intensity within UL44-positive vRC regions. To account for variations in DNA density, fluorescein-12-dATP signals were normalized to the corresponding DAPI intensity within the same ROI (mean ± SEM, n = 20 cells per condition from three independent experiments). Unpaired t-test with Welch’s correction. Scale bar, 10 μm.

MORC2 contains an N-terminal ATPase domain that is involved in regulating chromatin accessibility [30,32]. To assess whether MORC2 ATPase activity contributes to increased viral chromatin accessibility, we generated a recombinant HCMV expressing 3xFLAG-tagged ATPase-dead MORC2 mutant (MORC2 N39A HCMV) (S4A Fig). We first confirmed that the N39A mutation did not alter the subcellular localization of MORC2, as the mutant protein remained enriched in vRCs similarly to MORC2 WT (S4B Fig). We then evaluated the replication phenotypes of the recombinant viruses alongside parental WT HCMV. MORC2 WT HCMV exhibited increased viral genome replication and viral protein accumulation relative to parental WT HCMV. In contrast, MORC2 N39A HCMV showed viral DNA and protein levels comparable to those of the parental virus (Fig 3C and S4C Fig). Infectious progeny production followed a similar pattern. MORC2 WT HCMV produced significantly more infectious progeny than parental WT HCMV, whereas MORC2 N39A HCMV yielded progeny levels comparable to those of the parental virus (Fig 3D). Thus, in the presence of endogenous MORC2, the N39A mutant fails to confer the increased viral replication observed with MORC2 WT overexpression. Together, these findings indicate that the pro-viral effect of MORC2 overexpression depends on its ATPase activity.

Next, to determine whether MORC2 ATPase activity contributes to viral chromatin accessibility, we compared viral chromatin accessibility between cells infected with MORC2 WT HCMV and MORC2 N39A HCMV using a nuclease-based chromatin accessibility assay. Viral chromatin accessibility was significantly lower in cells infected with MORC2 N39A HCMV than in those infected with MORC2 WT HCMV (Fig 3E). To validate this finding using an orthogonal approach and to visualize accessible chromatin within vRCs, we used NicE-view labeling, an imaging-based chromatin accessibility assay that detects accessible DNA through incorporation of fluorescein-12-dATP [49]. We quantified NicE-view signals by normalizing fluorescein intensity to DAPI intensity within UL44-positive vRC regions. Consistent with the qPCR-based assay, cells infected with the MORC2 N39A HCMV displayed markedly reduced fluorescein signal within vRCs compared with those infected with the MORC2 WT HCMV, indicating reduced viral chromatin accessibility (Fig 3F).

### HCMV UL112-113 associates with MORC2 and promotes its enrichment within UL112-113 condensates

We next examined the mechanism underlying MORC2 enrichment within vRCs. MORC2 was enriched in UL44-positive pre-vRCs as early as 24 hpi (Fig 1B) and remained colocalized with UL44 following ganciclovir treatment (Fig 4A), suggesting that robust viral DNA synthesis is not required for MORC2 enrichment in vRCs. We therefore considered whether UL112-113 might mediate this process. Recently, UL112-113 has been identified as a key organizer of pre-vRC formation that forms nuclear condensates via LLPS and recruits UL44 to pre-vRCs[13]. To investigate the association between UL112-113 and MORC2 during infection, we generated a recombinant HCMV in which UL112-113 was N-terminally V5-tagged at its native viral locus (V5-UL112-113 HCMV) (Fig 4B and S5A Fig). To verify that V5 tagging did not affect viral replication, we compared the recombinant virus with the parental WT virus. Immunoblot analysis detected four V5-tagged UL112-113 isoforms specifically in cells infected with V5-UL112-113 HCMV, while viral protein accumulation was comparable to that observed with WT HCMV (S5B Fig). Infectious progeny production was also comparable between the two viruses (S5C Fig). Immunofluorescence analysis further showed that V5-UL112-113 localized to vRCs and colocalized with endogenous MORC2 throughout infection (24–72 hpi) (Fig 4C and S5D Fig).

**Fig 4:**
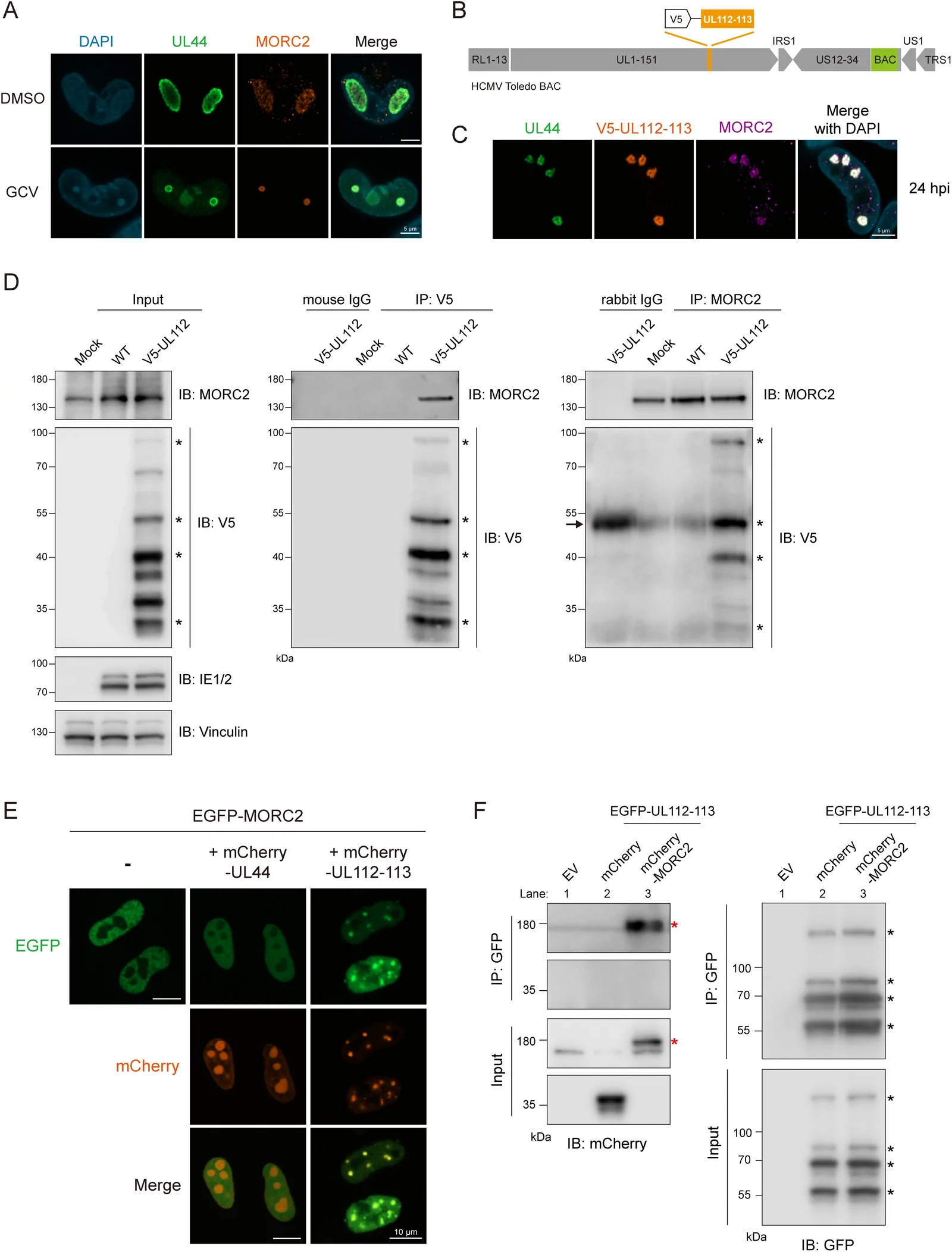
HCMV UL112-113 associates with MORC2 and colocalizes with it in viral replication compartments. **(A)** Representative confocal images demonstrate that MORC2 colocalizes with UL44-positive vRCs with or without GCV treatment (20 µM). HFFs were infected with WT HCMV (MOI = 1). At 48 hpi, cells were fixed and immunostained for UL44 (green) and MORC2 (orange). Nuclei were counterstained with DAPI (light blue). Scale bar, 5 µm. **(B)** Schematic representation of recombinant V5-UL112-113 HCMV BAC. The V5-tag was inserted into the N-terminal region of UL112-113 followed by a 6xGGS linker. **(C)** Immunofluorescence analysis of HFFs infected with V5-UL112-113 HCMV (MOI = 1) and fixed at 24 hpi. UL44 (green), V5-UL112-113 (orange), MORC2 (violet) and DAPI (light blue) are shown. Scale bar, 5 μm. **(D)** Co-IP analysis of UL112-113 and MORC2. HFFs were mock-infected or infected with WT or V5-UL112-113 HCMV (MOI = 3). In the figure, V5-UL112-113 HCMV is abbreviated as V5-UL112 for clarity. Cell lysates (Input) were subjected to immunoprecipitation using IgG, V5 or MORC2 antibody, followed by immunoblotting with the indicated antibodies. Vinculin served as a loading control for the input. Black asterisks indicate UL112-113 isoforms (p84, p50, p43, and p34), and the black arrow indicates a nonspecific band. Data are representative of two independent biological replicates. **(E)** Live-cell imaging of HeLa cells expressing EGFP-MORC2 alone or co-expressing EGFP-MORC2 with mCherry-UL44 or mCherry-UL112-113. Representative EGFP (green), mCherry (orange), and merged images are shown. Scale bar, 10 μm. **(F)** Co-IP assay in HeLa cells co-transfected with EGFP-UL112-113 and mCherry-MORC2 (or mCherry control). Cell lysates were subjected to immunoprecipitation using GFP nanobody-based beads. Input and IP samples were immunoblotted with anti-mCherry and anti-GFP antibodies, as indicated. The red asterisk indicates mCherry-MORC2, and black asterisks indicate UL112-113 isoforms (p84, p50, p43, and p34). EV: empty vector. Data are representative of two independent biological replicates.

To determine whether endogenous MORC2 associates with UL112-113 during infection, we performed reciprocal co-immunoprecipitation (co-IP) using lysates from mock-infected cells and cells infected with WT or V5-UL112-113 HCMV. Anti-V5 IP recovered endogenous MORC2 specifically from V5-UL112-113 HCMV-infected cells, whereas no corresponding MORC2 signal was detected in mock-infected, WT-infected, or IgG control samples. Reciprocally, anti-MORC2 IP recovered V5-tagged UL112-113 species specifically from V5-UL112-113 HCMV-infected cells, but not from mock-infected, WT-infected, or IgG control samples (Fig 4D). Together, these co-IP experiments support an association between endogenous MORC2 and UL112-113 in the context of HCMV infection.

Because numerous viral and host proteins are present within vRCs during HCMV infection, we next asked whether UL112-113 alone could promote MORC2 recruitment. To address this question, we co-expressed EGFP-MORC2 with mCherry-UL112-113 in HeLa cells. As a control, we used UL44, a viral DNA polymerase processivity factor and a major component of pre-vRC and RC structures [13, 50]. While EGFP-MORC2 alone displayed a diffuse pan-nuclear distribution (left panel), co-expression with mCherry-UL112-113 induced discrete nuclear condensates that robustly concentrated EGFP-MORC2 (right panel). In contrast, co-expression with mCherry-UL44 failed to recruit EGFP-MORC2 into UL44-positive foci (middle panel), suggesting that MORC2 recruitment is not a general property of vRC-associated viral proteins but is preferentially promoted by UL112-113 (Fig 4E). We next asked whether the physical association between UL112-113 and MORC2 could be recapitulated in an ectopic expression system in the absence of other viral proteins. In a co-IP assay, EGFP-UL112-113 pulled down mCherry-MORC2, while mCherry alone did not co-precipitate, supporting an association between UL112-113 and MORC2 (Fig 4F). Collectively, these findings indicate that UL112-113 associates with MORC2 and promotes its enrichment within UL112-113-driven nuclear condensates.

### Phase separation of UL112-113 facilitates MORC2 localization to viral replication compartments

To investigate whether UL112-113 phase separation facilitates MORC2 recruitment, we first used a simplified non-infection system in HeLa cells and then examined the sensitivity of MORC2 localization within UL112-113-driven condensates to hexanediol-mediated disruption. Because LLPS is a highly dynamic and reversible process, we monitored the localization of UL112-113 and MORC2 by live-cell imaging. When mCherry-UL112-113 and EGFP-MORC2 were co-expressed in HeLa, the two proteins strongly colocalized within nuclear foci. These foci were rapidly dispersed upon treatment with 1,6-hexanediol (1,6-HD), a chemical known to disrupt the weak hydrophobic interactions essential for LLPS [51] (Fig 5A). In contrast, treatment with 2,5-hexanediol (2,5-HD), a structural isomer used as a negative control, failed to dissolve the foci (Fig 5B). We also tested if UL112-113-MORC2 association depends on phase-separated condensate formation. HeLa cells were co-transfected with EGFP-UL112-113 and mCherry-MORC2 and treated with either 2,5-HD or 1,6-HD prior to lysis (Fig 5C). Consistent with the imaging results, co-IP analysis showed that 1,6-HD treatment reduced the association between UL112-113 and MORC2, whereas 2,5-HD had no detectable effect (Fig 5D). These data suggest that condensate formation promotes association between UL112-113 and MORC2 in the absence of other viral proteins and viral DNA.

**Fig 5:**
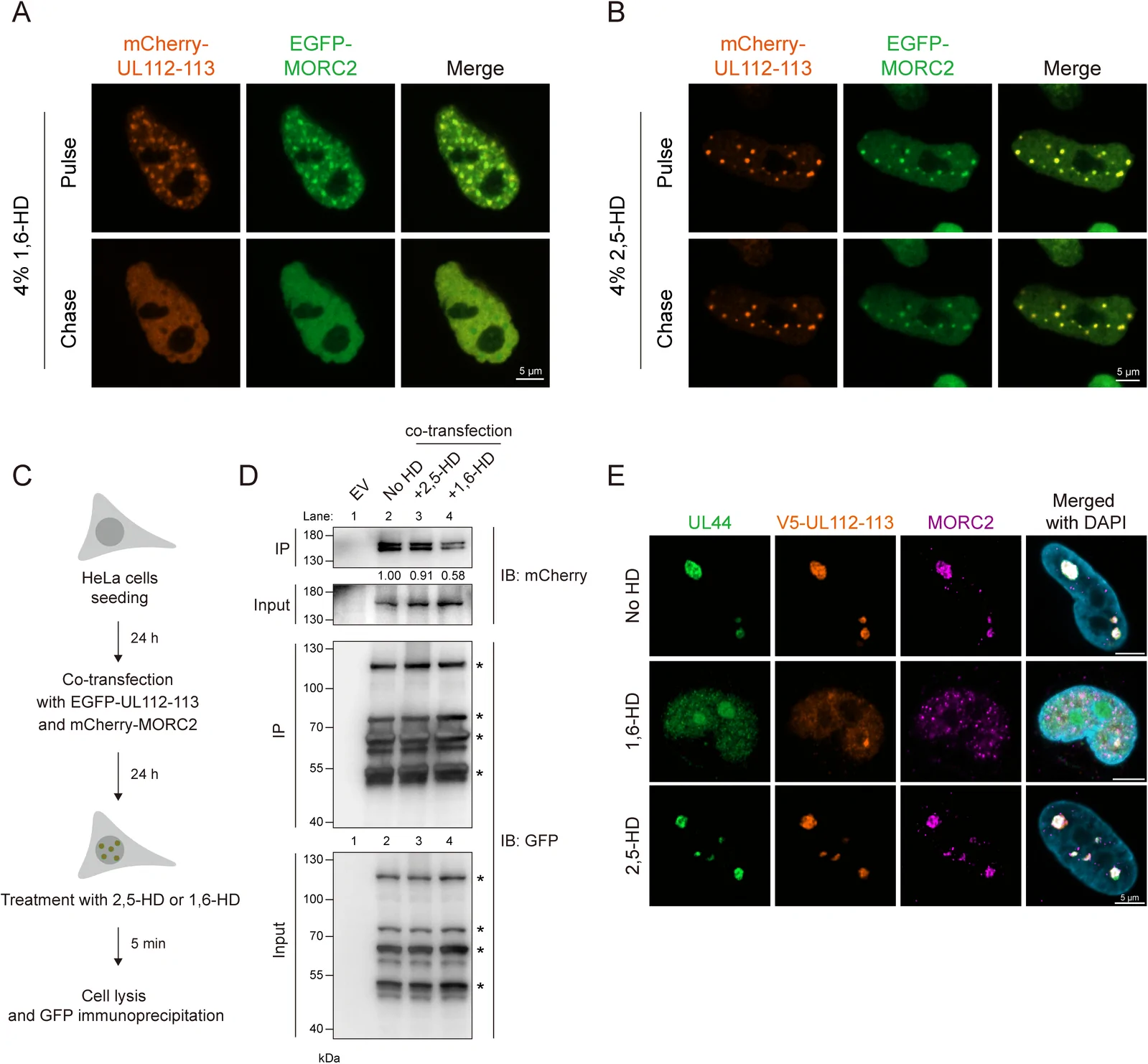
UL112-113 phase separation promotes MORC2 enrichment in viral replication compartments. **(A and B)** Live-cell imaging of HeLa cells co-expressing mCherry-UL112-113 and EGFP-MORC2 before (Pulse) and after (Chase) treatment for 5 min with 4% 2,5-HD (w/v) or 4% 1,6-HD (w/v). Representative images are shown. Scale bar, 5 μm. **(C)** Schematic of the experimental workflow to assess whether the UL112-113-MORC2 association depends on phase separation. **(D)** Co-IP assay in HeLa cells co-transfected with EGFP-UL112-113 and mCherry-MORC2 and treated with 4% 2,5-HD (w/v) or 4% 1,6-HD (w/v). Input and IP samples were immunoblotted with anti-mCherry and anti-GFP antibodies, as indicated. Black asterisks indicate four isoforms of UL112-113 (p84, p50, p43, p34). EV: empty vector. Data are representative of two independent biological replicates. **(E)** Immunofluorescence assay in HFFs infected with V5-UL112-113 HCMV (MOI = 1). At 24 hpi, cells were treated with 2% 2,5-HD (w/v) or 2% 1,6-HD (w/v) for 5 min, followed by staining for UL44 (green), V5-UL112-113 (orange), MORC2 (violet), and DAPI (light blue). Scale bar, 5 μm.

Next, we examined whether MORC2 enrichment within UL112-113-containing condensates is sensitive to perturbation of condensate organization during HCMV infection. HFFs were infected with the V5-UL112-113 HCMV, and the localization of endogenous MORC2 was analyzed together with UL44 and V5-UL112-113. In untreated cells, V5-UL112-113 formed discrete nuclear structures within UL44-positive vRCs, where endogenous MORC2 was also prominently enriched. Treatment with 1,6-HD disrupted the punctate organization of V5-UL112-113 and was accompanied by a marked redistribution of MORC2 from these structures. By contrast, treatment with 2,5-HD did not appreciably alter the localization of either V5-UL112-113 or MORC2 under the conditions tested (Fig 5E). These observations indicate that the enrichment of MORC2 within UL112-113-containing vRCs is sensitive to perturbation of condensate organization during infection. Together, these results support a model in which UL112-113 phase separation promotes its association with MORC2 and facilitates MORC2 enrichment within viral replication compartments.

### The MORC2 CC3 domain contributes to MORC2 association with UL112-113 and localization to viral replication compartments

We next sought to identify the MORC2 domains responsible for its association with UL112-113. MORC2 consists of an N-terminal ATPase domain, coiled-coil 1 (CC1), an S5 domain, a CW-type zinc finger (CW-ZF), coiled-coil 2 (CC2), a chromo-like domain (CD), and coiled-coil 3 (CC3) (Fig 6A). Because UL112-113 forms phase-separated condensates and intrinsically disordered regions (IDRs) often contribute to protein recruitment into biomolecular condensates [52,53], we first analyzed the MORC2 amino acid sequence using the IDR prediction algorithm (IUPred3) [54]. Using a disorder score threshold of 0.5, we identified amino acids 523–799 as a predicted IDR that overlaps the CC2 domain and contains the previously reported NLSs [55] (Fig 6A and S6A Fig). To map the MORC2 regions required for association with UL112-113, we generated a series of mCherry-tagged MORC2 deletion mutants, including a mutant lacking the predicted IDR. Because deletion of the IDR also removed the endogenous NLSs, an SV40 NLS was fused to this mutant to preserve its nuclear localization (S6B Fig).

**Fig 6:**
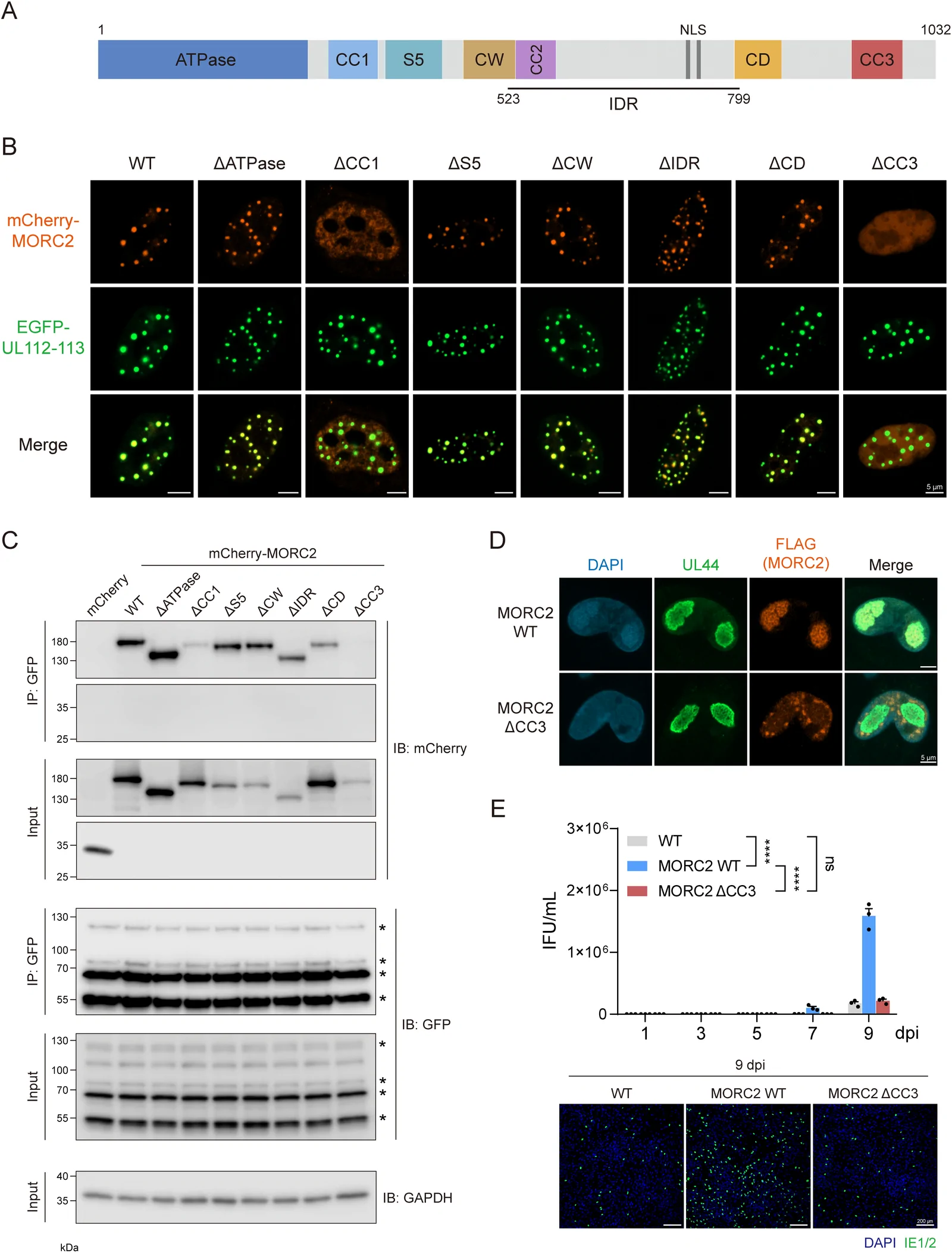
CC3 domain deletion impairs MORC2 association with UL112-113 and recruitment to viral replication compartments. **(A)** Schematic representation of MORC2 domain organization, the predicted IDR, and NLSs. Domain boundaries are indicated as follows: ATPase, aa 1-265; CC1, aa 282-362; S5, aa 367-469; CW-type zinc finger (CW), aa 493-544; CC2, aa 547-584; chromo-like domain (CD), aa 794-854; and CC3, aa 966-1016. The predicted IDR spans aa 523-799, and the NLSs are located at aa 734-742 and 755-771. **(B)** Live-cell imaging of HeLa cells co-expressing mCherry-MORC2 WT or domain deletion mutants and EGFP-UL112-113. Representative images are shown. Scale bar, 5 μm. **(C)** Co-IP assay in HeLa cells co-transfected with EGFP-UL112-113 and mCherry-MORC2 WT, the indicated MORC2 deletion mutants, or mCherry control. EGFP-UL112-113 was pulled down using GFP-Trap. Input and IP samples were immunoblotted with the indicated antibodies. Black asterisks indicate four isoforms of UL112-113 (p84, p50, p43, p34). Data are representative of two independent biological replicates. **(D)** Immunofluorescence assay in HFFs infected with MORC2 WT or MORC2 ΔCC3 HCMV (MOI = 1). At 48 hpi, cells were fixed, followed by staining for UL44 (green), FLAG (orange), and DAPI (light blue). Scale bar, 5 μm. **(E)** Top, cell-free progeny virus in the supernatant was quantified by limiting dilution analysis at the indicated time points from HFFs infected with WT, MORC2 WT, or MORC2 ΔCC3 HCMV (MOI = 0.1) (mean ± SEM, n = 3). Bottom, representative immunofluorescence images of fresh HFFs infected with 10-fold diluted cell-free supernatants collected at 9 dpi. Cells were stained for IE1/2 (green) and counterstained with DAPI (blue). Two-way ANOVA with Sidak’s multiple comparisons test was performed across the time course; statistical comparisons at 9 dpi are shown. Scale bar, 200 μm.

We next co-expressed each mCherry-tagged MORC2 mutant with EGFP-UL112-113 in HeLa cells and examined their subcellular localization by live-cell imaging. Deletion of most MORC2 regions examined, including the predicted IDR, did not appreciably affect MORC2 enrichment within UL112-113 condensates. In contrast, deletion of CC1 partially reduced MORC2 enrichment, whereas deletion of CC3 almost completely abolished condensate localization, resulting in a diffuse pan-nuclear distribution (Fig 6B). To further characterize the effects of these domain deletions, we examined MORC2 association with UL112-113 by co-IP. Although reduced recovery was also observed for ΔCC1 and ΔCD, ΔCC3 showed the most pronounced reduction among the deletion mutants (Fig 6C). Together, these results indicate that the CC3 domain is particularly important for both MORC2 recruitment to UL112-113 condensates and its association with UL112-113.

Given the pronounced effect of CC3 deletion in the ectopic expression system, we next examined whether this domain also contributes to MORC2 recruitment into vRCs during HCMV infection. To this end, we generated a recombinant HCMV expressing 3xFLAG-tagged MORC2 lacking the CC3 domain (MORC2 ΔCC3 HCMV) (S6C Fig). We first assessed the subcellular localization of MORC2 ΔCC3 during infection. MORC2 WT was enriched within UL44-positive vRCs, whereas MORC2 ΔCC3 was distributed throughout the nucleus in discrete puncta and showed little enrichment within vRCs (Fig 6D). To assess the functional significance of the reduced vRC recruitment observed with MORC2 ΔCC3, we next compared infectious progeny production among WT HCMV, MORC2 WT HCMV, and MORC2 ΔCC3 HCMV. MORC2 WT HCMV produced more infectious progeny than parental WT HCMV, whereas MORC2 ΔCC3 HCMV showed progeny production comparable to that of parental WT HCMV (Fig 6E). Together, these findings indicate that the CC3 domain is important for MORC2 recruitment to vRCs and suggest a functional link between efficient MORC2 recruitment to vRCs and enhanced HCMV replication.

## Discussion

In this study, we uncover a novel mechanism by which HCMV hijacks the host chromatin remodeler MORC2 to promote viral DNA replication. We show that HCMV induces MORC2 expression and that UL112-113-mediated phase separation facilitates MORC2 recruitment to vRCs. Within vRCs, MORC2 promotes DDR activation and the accumulation of DNA repair factors, thereby alleviating replication stress and facilitating productive infection.

DDR is a critical mechanism for the maintenance of genome integrity. Efficient genome replication requires active DNA repair mechanisms, particularly for viruses with large genomes such as herpesviruses. Indeed, depletion of host DDR proteins that localize within vRCs, such as ATM, has been shown to impair HCMV replication [18], showing that the virus must actively utilize host DNA repair proteins for replication. Generally, DNA viruses are considered to maintain higher genomic stability compared to RNA viruses [56,57] but recent studies have highlighted that HCMV exhibits a surprisingly high mutation rate and genomic diversity comparable to RNA viruses [58,59]. By recruiting a clade of different repair machineries which partake in both error-prone and high-fidelity DNA repair pathways by sequestering the upstream factor MORC2 to vRC, HCMV may achieve efficient DNA replication with added evolutionary advantage. Recent studies have revealed intricate manipulation of the Fanconi anemia repair pathway by UL138 protein, the product of an early-late gene, and recruitment of low-fidelity translesion synthesis DNA polymerases to the HCMV vRC [60,61]. It would be interesting to test if the initial recruitment of the host factors involved in these alternative DNA repair and synthesis pathways also relies on MORC2 activity.

Several studies have documented canonical DDR activation during herpesvirus infection, including ATM/ATR signaling and checkpoint modulation [18,62–65]. Importantly, accumulating evidence indicates that DDR factors are not merely activated but are spatially reprogrammed to vRCs, where they can act on replicating viral genomes. For instance, during HSV-1 infection, ATM and cellular mismatch proteins MSH2/MLH1 accumulate at vRCs, and these factors are required for efficient HSV-1 replication [66,67]. Similarly, in EBV and KSHV, various repair factors ranging from the MRN complex to homologous recombination proteins like RAD51 are preferentially recruited to replicating viral genomes within vRCs [68, 69]. Although these studies suggest that herpesviruses can direct DDR to preferentially act on viral DNA, the mechanism by which DDR factors are relocalized to vRCs of these other herpesviruses remains to be elucidated.

LLPS is a biophysical process through which proteins and nucleic acids condense into dynamic, membraneless compartments that enable spatial and temporal regulation of cellular processes [14,70]. Just as host cells utilize LLPS to concentrate repair machineries [71], our findings suggest that HCMV exploits a similar strategy. In this model, UL112-113-driven condensates serve as a scaffold that facilitates MORC2 recruitment. This spatial confinement of MORC2 within UL112-113 droplets allows for the modulation of the chromatin environment around the viral DNA, thereby facilitating DDR signaling. Thus, UL112-113 condensates provide a virus-driven platform that couples host factor recruitment with the induction of DDR signaling essential for viral replication. Interestingly, recent studies have shown that HSV-1 encodes ICP4, which undergoes LLPS and contributes to the formation of replication compartments during infection [72,73]. Our finding that MORC2 is recruited into UL112-113 condensates raises the possibility that herpesviruses more broadly exploit LLPS-based mechanisms to recruit host factors involved in DDR and to selectively coordinate their functions for productive viral replication.

Our domain-mapping analysis further provides insight into the molecular basis of MORC2 recruitment to UL112-113 condensates. A previous study has shown that UL112-113 forms nuclear condensates and recruits UL44 into pre-replication compartments, with the intrinsically disordered region (IDR) of UL44 contributing to this recruitment [13]. Based on this finding, we initially considered whether the predicted IDR of MORC2 might similarly contribute to its recruitment into UL112-113 condensates. Unexpectedly, deletion of the MORC2 IDR had little effect on either its enrichment within UL112-113 condensates or its association with UL112-113. In contrast, CC3 deletion produced the most pronounced effects on both MORC2 enrichment within UL112-113 condensates and association with UL112-113, whereas CC1 deletion caused partial defects (Fig 6B and 6C). CC1 has been implicated in the DNA-binding activity of MORC2 [74], whereas CC3 has been implicated in MORC2 dimerization [55,75]. These distinct properties raise the possibility that CC1 and CC3 may contribute to MORC2 association with UL112-113 and condensate recruitment through different mechanisms. Whether and how these domain-specific activities contribute to MORC2 association with UL112-113 and condensate recruitment remains to be determined.

Collectively, our findings support a model in which HCMV promotes the enrichment of MORC2 within viral replication compartments through its association with UL112-113-containing condensates. Once recruited, MORC2 ATPase activity may enhance viral chromatin accessibility and facilitate DDR factor accumulation, thereby creating an environment favorable for efficient viral DNA replication. This model provides a framework for understanding how HCMV coordinates host chromatin remodeling and DNA damage responses during lytic infection.

## Materials and methods

### Cells

Primary HFFs, MRC-5, U373MG and HeLa cells were maintained in Dulbecco’s modified Eagle’s medium (DMEM; HyClone) supplemented with 10% fetal bovine serum (FBS; HyClone) and 1% (v/v) GlutaMAX-I (Gibco). Cells were grown at 37°C under 5% CO_2_ atmosphere.

### Viruses

HCMV Toledo and TB40/E BAC DNAs were gifts from Thomas E. Shenk (Princeton University) and Eain Murphy (Upstate Medical University), respectively. Unless otherwise indicated, all HCMV experiments were performed using the Toledo strain or Toledo BAC-derived recombinant viruses. TB40/E was used only in the experiments explicitly indicated. All experiments involving HCMV were conducted in accordance with protocols approved by the Institutional Biosafety Committee of Seoul National University (SNUIBC-R181130-2). Infectious HCMV was generated by electroporation of BAC DNA into primary HFFs using the Neon Transfection System (MPK5000; Invitrogen) at 1,700 V for 20 ms with 1 pulse. Upon reaching complete cytopathic effect, culture supernatants were harvested, and virions were pelleted by centrifugation at 40,000 × g for 1 h at 4°C, followed by resuspension in serum-free DMEM. For UV inactivation, virus stocks were placed on ice and irradiated with UV light at 300 mJ/cm^2^.

### RNA interference

ON-TARGETplus SMARTpool or individual siRNAs targeting MORC2, 53BP1, and RAD51, as well as non-targeting controls, were purchased from Dharmacon. All transfections of siRNA were conducted using DharmaFECT 1 transfection reagent (Dharmacon).

### RNA extraction and quantitative reverse transcription PCR (qRT-PCR)

Total RNA was extracted using the NucleoSpin RNA kit (740955.250; Macherey-Nagel) according to the manufacturer’s instructions. 1 μg of total RNA was reverse-transcribed into cDNA using ReverTra Ace qPCR RT Master Mix (FSQ-101; Toyobo) according to the manufacturer’s protocol. Quantitative PCR was performed using TOPreal qPCR PreMIX (RT500M; Enzynomics) with the iCycler iQ Real-Time PCR Detection System (Bio-Rad). Gene expression levels were normalized to GAPDH. All primers used for qPCR are listed in S1 Table.

### Generation of recombinant HCMV

To generate recombinant HCMV expressing MORC2 WT-3xFLAG, the HCMV Toledo BAC was modified using a counter-selection BAC modification kit (GeneBridge) according to the manufacturer’s instructions. Briefly, the 102-bp intergenic region between US1 and US2 in the Toledo BAC was first replaced with an rpsL-neoR selection cassette by homologous recombination. The prokaryotic selection cassette was then replaced with a PCR-amplified CMV-MORC2 WT-3xFLAG cassette. MORC2 N39A-3xFLAG and MORC2 ΔCC3-3xFLAG HCMV BACs were subsequently generated from the MORC2 WT-3xFLAG HCMV BAC by introducing the N39A point mutation or deleting the CC3 domain, respectively, using the same counter-selection strategy. To generate V5-UL112-113 HCMV, the endogenous UL112-113 locus in the Toledo BAC was modified to introduce an N-terminal V5 tag followed by a 6xGGS linker using the same counter-selection strategy. The sequences of the homology arms used for each BAC recombination are provided in S2 Table.

Correct recombination was initially assessed by PCR. For the MORC2 WT-3xFLAG HCMV BAC, the entire inserted cassette together with approximately 500 bp of flanking viral genomic sequence on each side was verified by Sanger sequencing. For the MORC2 N39A-3xFLAG and MORC2 ΔCC3-3xFLAG HCMV BACs, which were generated from the verified MORC2 WT-3xFLAG HCMV BAC, the entire MORC2-3xFLAG cassette was sequenced to confirm the N39A mutation or CC3 deletion, respectively. For V5-UL112-113 HCMV, the modified N-terminal region of UL112-113, including the V5-6xGGS insertion and approximately 500 bp of flanking viral genomic sequence on each side, was verified by Sanger sequencing. BAC DNA was subsequently purified using the NucleoBond Xtra BAC kit (740436; Macherey-Nagel) and analyzed by restriction enzyme digestion followed by agarose gel electrophoresis and ethidium bromide staining. The verified BAC DNAs were then transfected into primary HFFs to reconstitute infectious virus.

### Immunoblotting

Cells were harvested and lysed in RIPA buffer (50 mM Tris-HCl, pH 7.4, 150 mM NaCl, 0.5% sodium deoxycholate, 0.1% SDS, and 1.0% NP-40) supplemented with 1 mM dithiothreitol (DTT), 1 mM phenylmethanesulfonyl fluoride (PMSF), and 1× Protease Inhibitor Cocktail (11836170001; Roche). Lysates were mixed with Laemmli sample buffer, boiled at 98°C for 10 min, and separated on Novex Tris-Glycine gels (Invitrogen). Proteins were transferred onto nitrocellulose or PVDF membranes. Membranes were blocked in 5% skim milk in TBST for 1 h at room temperature and then incubated with the indicated primary antibodies overnight at 4°C. Primary antibodies used in this study were as follows: mouse anti-vinculin (V9131; Sigma-Aldrich), mouse anti-HSC70 (ab2788; Abcam), rabbit anti-GAPDH (LF-PA0212; Ab Frontier), rabbit anti-53BP1 (ab175933; Abcam), rabbit anti-RAD51 (ab133534; Abcam), rabbit anti-MORC2 (A300-149A; Bethyl Laboratories), mouse anti-IE1/2 (MAB810R; Merck), mouse anti-UL44 (CA006; Virusys), mouse anti-pp28 (CA004; Virusys), mouse anti-FLAG M2 (F3165; Sigma-Aldrich), mouse anti-V5 (R960-25; Invitrogen), rabbit anti-V5 (13202T; Cell Signaling Technology), mouse anti-GFP (sc-9996; Santa Cruz Biotechnology), and rabbit anti-mCherry (43590; Cell Signaling Technology). Membranes were washed three times with TBST for 10 min each and incubated with horseradish peroxidase-conjugated secondary antibodies diluted 1:5,000 in 5% skim milk for 1 h at room temperature. Immunoreactive bands were visualized using SuperSignal West Pico PLUS or Atto Ultimate Sensitivity chemiluminescent substrates (34580 or A38555; Thermo Fisher Scientific).

### Immunofluorescence assay

Cells were seeded onto 18-mm coverslips in 12-well plates for imaging. Cells were washed twice with PBS and fixed with 3.7% formaldehyde for 10 min at room temperature. For FLAG and RAD51 imaging, a pre-extraction step was performed to reduce cytosolic background signals, with minor modifications from a previously described protocol [17], followed by two washes with PBS and fixation with 3.7% formaldehyde for 10 min at room temperature. After fixation, cells were washed twice with PBS and permeabilized with 0.1% Triton X-100 in PBS. Cells were then washed with blocking solution (2% BSA in PBS) and incubated with primary antibodies overnight at 4°C. Primary antibodies used in this study were as follows: rabbit anti-MORC2 (NBP1-89295; Novus Biologicals), goat anti-V5 (A190-119A; Bethyl Laboratories), goat anti-FLAG (NB600-344; Novus Biologicals), mouse anti-IE1/2 (MAB810R; Merck), mouse anti-UL44 (CA006; Virusys), rabbit anti-γH2AX (9718; Cell Signaling Technology), rabbit anti-53BP1 (ab175933; Abcam), rabbit anti-RAD51 (ab133534; Abcam), mouse anti-BrdU (5292; Cell Signaling Technology). Following primary antibody incubation, cells were washed twice with blocking solution and incubated with fluorophore-conjugated secondary antibodies for 1 h at room temperature. After secondary antibody incubation, cells were washed twice with blocking solution and once with PBS. Finally, coverslips were mounted onto glass slides using VECTASHIELD antifade mounting medium containing DAPI (H-1200, Vector Laboratories). Images were acquired using LSM700 or LSM980 confocal laser-scanning microscopes (ZEISS).

For GCV treatment experiments, HFFs were infected with WT HCMV at an MOI of 1. After 1 h of infection, the virus-containing medium was replaced with fresh medium containing GCV (20 μM) or an equivalent volume of DMSO. Cells were fixed at 48 hpi and processed for immunofluorescence analysis as described above.

For hexanediol treatment experiments, HFFs were infected with V5-UL112-113 HCMV at an MOI of 1. At 24 hpi, cells were treated with 2% (w/v) 1,6-hexanediol (1,6-HD, 240117; Sigma-Aldrich) or 2% (w/v) 2,5-hexanediol (2,5-HD, H11904; Sigma-Aldrich) for 5 min at 37°C. Cells were then washed with PBS and processed for immunofluorescence analysis as described above.

### Quantification of HCMV DNA copy number

Total DNA was extracted using the QIAamp DNA Blood Mini Kit (51106; Qiagen) according to the manufacturer’s instructions. HCMV genome copy number was determined using primers targeting UL86, with 7SK used as an internal normalization control. A standard curve was generated using serial dilutions of HCMV Toledo BAC DNA (2 pg to 2 ng) supplemented with HFF genomic DNA to maintain a constant total DNA input of 20 ng.

### Viral progeny production assay

HFFs were infected with HCMV at an MOI of 0.1, and cell-free supernatants were collected at the indicated time points. To quantify infectious progeny, fresh HFFs were infected with the harvested supernatants and fixed the following day with 3.7% formaldehyde. Cells were then permeabilized with 0.1% Triton X-100 and blocked with 2% BSA in PBS. Infected cells were detected by incubation with anti-IE1/2 antibody overnight at 4°C, followed by three washes with blocking solution and incubation with Alexa Fluor 488-conjugated anti-mouse secondary antibody for 1 h at room temperature. After two washes with blocking solution and one wash with PBS, nuclei were counterstained with DAPI in PBS.

### EdU/BrdU dual-labeling assay and calculation of colocalization coefficient

Cells were treated with siRNAs and seeded onto 18-mm coverslips in 12-well plates for imaging. Cells were infected with HCMV at an MOI of 1. At 48 hpi, EdU was added at a final concentration of 1 μM for 16 h to label replicating viral DNA. During the final 1 h of EdU labeling, cells were treated with 1 μM camptothecin (CPT). After removal of the medium, BrdU was added at a final concentration of 10 μM for 5 h to monitor replication restart. Cells were then washed twice with PBS, fixed with 3.7% formaldehyde in PBS for 10 min at room temperature, and permeabilized with 0.1% Triton X-100 in PBS for 10 min. After blocking with 2% BSA in PBS, incorporated EdU was detected by click chemistry using the Click-iT Plus EdU Alexa Fluor 488 Imaging Kit (C10637; Thermo Fisher Scientific) according to the manufacturer’s instructions. Cells were subsequently incubated with BrdU antibody (5292; Cell Signaling Technology) diluted in blocking solution overnight at 4°C, washed three times with blocking solution, and incubated with Alexa Fluor 568-conjugated goat anti-mouse antibodies (A-11004; Invitrogen) diluted in blocking solution for 1 h at room temperature. After two additional washes with blocking solution and one wash with PBS, coverslips were mounted onto glass slides using VECTASHIELD antifade mounting medium containing DAPI. Images were acquired using an LSM 700 confocal laser-scanning microscope (ZEISS). Colocalization was quantified in ZEN Blue (ZEISS) by measuring the number of pixels in channel 1 overlapping with those in channel 2 within each cell.

### Co-immunoprecipitation

For co-immunoprecipitation experiments using infected cells, HFFs were mock-infected or infected with parental WT HCMV or V5-UL112-113 HCMV (MOI = 3). At 48 hpi, cells were harvested and lysed in lysis buffer [10 mM HEPES (pH 7.6), 150 mM KCl, 0.5% NP-40, 2 mM MgCl_2_, 10% glycerol, and 25 U/mL Benzonase] supplemented with 1 mM DTT, 1 mM PMSF, and 1× protease inhibitor cocktail. For V5 or MORC2 immunoprecipitation, mouse anti-V5 antibody (R960-25; Invitrogen) or rabbit anti-MORC2 antibody (A300-149A; Bethyl Laboratories) was first incubated with Protein G beads (10004D; Invitrogen) for 1 h at room temperature. The antibody-bound beads were then added to the cell lysates and incubated overnight at 4°C. Mouse IgG (sc-2025; Santa Cruz Biotechnology) and rabbit IgG (2729; Cell Signaling Technology) were used as isotype controls for anti-V5 and anti-MORC2 immunoprecipitation, respectively.

To examine the association between UL112-113 and MORC2 in HeLa cells, EGFP-UL112-113 was co-transfected with either mCherry-MORC2 or an mCherry control vector. Cells were harvested 24 h post-transfection and lysed in the same lysis buffer. EGFP-tagged proteins were immunoprecipitated using GFP-Trap magnetic agarose beads (gtma-20, ChromoTek) for 2 h at 4°C.

To assess the dependence of the UL112-113-MORC2 association on LLPS, HeLa cells co-transfected with EGFP-UL112-113 and mCherry-MORC2 were treated with 4% (w/v) 1,6-HD or 4% (w/v) 2,5-HD for 5 min immediately prior to lysis. Cell lysates were then subjected to GFP-Trap immunoprecipitation as described above.

Immune complexes were washed with NT2-K buffer [50 mM HEPES (pH 7.6), 150 mM KCl, 0.05% NP-40, and 2 mM EDTA] supplemented with 1 mM DTT, 1 mM PMSF, and 1× protease inhibitor cocktail. Bound proteins were eluted in Laemmli sample buffer and analyzed by immunoblotting. For input controls, 2% of the total cell lysate was loaded.

### Chromatin accessibility assay

Chromatin accessibility was assessed using the EpiQuik Chromatin Accessibility Assay Kit (P-1047-48; EpiGentek) according to the manufacturer’s instructions. Briefly, 2 × 10^6^ primary HFFs were seeded in 100-mm dishes and infected the following day with HCMV at an MOI of 1. At 48 hpi, 1 × 10^6^ cells were harvested and divided into two equal aliquots. Cells were lysed, and one aliquot was treated with nuclease (Nse+), whereas the other was left untreated (Nse−). DNA was then purified, and qPCR was performed using primer pairs targeting the indicated viral loci, with NANOG and ACTB included as controls. The Fold Enrichment (FE) was calculated using the following formula: *FE* = 2^(Ct(Nse+)-Ct(Nse-))^ × 100%.

### NicE-view assay

To analyze viral chromatin openness, we adapted a previously described protocol with minor modifications [49]. Briefly, 2 × 10^5^ primary HFFs were seeded onto coverslips and cultured to confluence. Cells were then infected with HCMV at an MOI of 1. At 48 hpi, cells were fixed with 1% formaldehyde for 10 min at room temperature and quenched with 125 mM glycine for 5 min. After two washes with PBS, cells were incubated in cytosolic lysis buffer for 10 min at 4°C. Following two additional washes with PBS, chromatin labeling was performed for 2 h at 37°C in a reaction buffer containing 1× NEBuffer 2, Nt.CviPII, DNA polymerase I, a dNTP mixture, and fluorescein-12-dATP. The reaction was terminated with stop solution containing RNase A, followed by one wash with PBS at 55°C and two additional washes at room temperature. For immunofluorescence staining, cells were incubated with mouse anti-UL44 primary antibody diluted in 2% BSA blocking solution overnight at 4°C. The following day, cells were washed three times with blocking solution and incubated with Alexa Fluor 568-conjugated anti-mouse secondary antibody for 1 h at room temperature. Coverslips were then mounted using VECTASHIELD antifade mounting medium containing DAPI.

### Live-cell imaging

For live-cell imaging, HeLa cells were seeded at a density of 1 × 10^5^ cells per dish in 35-mm glass-bottom confocal dishes. The following day, the cells were transfected with fluorescently tagged protein expression vectors using Lipofectamine 3000 (Invitrogen) according to the manufacturer’s protocol. At 24 h post transfection, the culture medium was replaced with pre-warmed phenol red-free live-imaging medium to minimize background autofluorescence. To assess the LLPS properties, the medium was replaced with pre-warmed medium containing 4% (w/v) 1,6-HD or 2,5-HD, and the dissolution of mCherry-UL112-113 and EGFP-MORC2 foci was monitored. Live-cell imaging was performed using a ZEISS LSM 980 confocal microscope equipped with an Airyscan 2 detector for enhanced sensitivity and resolution. To maintain physiological conditions, the cells were kept in a top-stage incubation chamber at 37°C with a humidified 5% CO2 atmosphere. Images were acquired using a 63× oil-immersion objective. Image processing and analysis were performed using ZEISS ZEN (blue edition) software.

### Statistical analysis

GraphPad Prism 7.00 (GraphPad Software) was used for all statistical analyses. Data are presented as mean ± SEM. For comparisons between two groups, two-sided unpaired Student’s t-tests were used, with Welch’s correction applied when variances were unequal. Non-parametric comparisons were performed using a two-sided Mann-Whitney test. For multiple comparisons, one-way or two-way analysis of variance (ANOVA) followed by Dunnett’s or Sidak’s multiple comparisons test was used, as indicated in the figure legends. For multiple unpaired comparisons, false discovery rate (FDR) correction was applied using the Benjamini, Krieger and Yekutieli method (Q = 5%). Statistical significance is indicated as follows: *P < 0.05, **P < 0.01, ***P < 0.001, ****P < 0.0001; ns, not significant.

## Supporting information

S1 Fig HCMV depends on MORC2 for efficient lytic replication. (A) Immunoblot analysis of MORC2 and viral proteins (IE1/2, UL44, pp28) in HFFs infected with WT or UV-inactivated TB40/E HCMV (MOI = 2) at the indicated time points. MORC2 levels were normalized to vinculin and are shown relative to those in mock-infected cells. Vinculin served as a loading control (n = 2). (B) qRT-PCR analysis of MORC2 mRNA levels in siCtrl- or siMORC2-transfected HFFs infected with Toledo HCMV (MOI = 1). Cells were harvested at the indicated time points (mean ± SEM, n = 3). Two-way ANOVA with Sidak’s multiple comparisons test. (C) Immunoblot analysis of MORC2 and viral proteins (IE1/2, UL44, pp28) in HFFs transfected with siCtrl or four independent MORC2-targeting siRNAs (#1-#4) and mock-infected or infected with Toledo HCMV (MOI = 1). Cells were harvested at 72 hpi. GAPDH served as a loading control. (D) Immunoblot analysis of MORC2 and viral proteins (IE1/2, UL44, pp28) in siCtrl- or siMORC2-transfected HFFs infected with TB40/E HCMV (MOI = 1). Cells were harvested at the indicated time points. Vinculin served as a loading control (n = 2). (E) Left, cell-free progeny virus in the supernatant was quantified by limiting dilution analysis at the indicated time points from siCtrl- or siMORC2-transfected HFFs infected with TB40/E HCMV (MOI = 0.1) (mean ± SEM, n = 3). Right, representative immunofluorescence images of fresh HFFs infected with 10-fold diluted cell-free supernatants collected at 9 dpi from siCtrl- or siMORC2-transfected HFFs. Cells were stained for IE1/2 (green) and counterstained with DAPI (blue). Two-way ANOVA with Sidak’s multiple comparisons test was performed across the time course; statistical comparisons at 7 and 9 dpi are shown. Scale bar, 200 μm. (F) Restriction fragment length polymorphism (RFLP) analysis of WT HCMV BAC and MORC2 WT HCMV BAC. BAC DNA was digested with EcoRI and resolved by agarose gel electrophoresis to assess genome integrity and correct recombination. The red asterisk indicates an additional fragment derived from insertion of the CMV-MORC2-3xFLAG cassette. (G) Immunofluorescence analysis of HFFs infected with MORC2 WT HCMV (MOI = 1) and fixed at the indicated time points. UL44 (green), FLAG (orange), and DAPI (light blue) are shown. Scale bar, 5 μm.
(TIF)

S2 Fig MORC2 supports HCMV protein expression across multiple cell types. (A) Immunoblot analysis of MORC2 and viral proteins (IE1/2, UL44, pp28) in siCtrl- or siMORC2-transfected MRC-5 cells infected with WT HCMV (MOI = 1) and harvested at the indicated time points. GAPDH served as a loading control (n = 2). (B) Immunoblot analysis of MORC2 and viral proteins (IE1/2, UL44, pp28) in siCtrl- or siMORC2-transfected U373MG cells infected with WT HCMV (MOI = 1) and harvested at the indicated time points. GAPDH served as a loading control (n = 2).
(TIF)

S3 Fig 53BP1 and RAD51 promote HCMV replication and recovery from replication stress. (A) Immunoblot analysis confirming the knockdown efficiency of MORC2, 53BP1, or RAD51 in HFFs transfected with the indicated siRNAs. HSC70 was used as a loading control. Lane labels: C, control siRNA; M, siMORC2; 53, si53BP1; R, siRAD51; D, double knockdown of 53BP1 and RAD51. (B) Immunoblot analysis of viral proteins (IE1/2, UL44, pp28) in HFFs transfected with the indicated siRNAs and infected with HCMV (MOI = 1). Cells were harvested at the indicated time points. Vinculin served as a loading control (n = 2). (C) Quantification of HCMV DNA copy number by qPCR in siCtrl-, si53BP1-, or siRAD51-transfected HFFs infected with WT HCMV (MOI = 1) and harvested at the indicated time points (mean ± SEM, n = 3). Two-way ANOVA with Dunnett’s multiple comparisons test. (D) Dual-pulse labeling assay to assess replication restart following CPT-induced replication stress. HFFs transfected with siCtrl, si53BP1, or siRAD51 were infected with WT HCMV (MOI = 1). At 48 hpi, cells were labeled with EdU (green) for 16 h, with CPT added during the final 1 h to induce replication stress, followed by BrdU (magenta) labeling to monitor resumption of DNA synthesis. Nuclei were counterstained with DAPI (blue). Replication recovery was quantified based on the BrdU/EdU colocalization coefficient (40 cells per condition from three independent experiments). One-way ANOVA with Dunnett’s multiple comparisons test. Scale bars, 5 μm.
(TIF)

S4 Fig MORC2 N39A retains localization to viral replication compartments. (A) RFLP analysis of WT HCMV BAC, MORC2 WT HCMV BAC, and MORC2 N39A HCMV BAC. BAC DNA was digested with EcoRI and resolved by agarose gel electrophoresis to assess genome integrity and correct recombination. The red asterisk indicates an additional fragment derived from insertion of the CMV-MORC2 WT-3xFLAG or CMV-MORC2 N39A-3xFLAG cassette. (B) Immunofluorescence analysis of HFFs infected with MORC2 N39A HCMV (MOI = 1). Cells were fixed at 48 hpi and stained with anti-UL44 (green) and anti-FLAG (orange); nuclei were counterstained with DAPI (light blue). Scale bars, 10 µm. (C) Immunoblot analysis of MORC2, FLAG, and viral proteins (IE1/2, UL44, pp28) in mock-infected HFFs or HFFs infected with parental WT, MORC2 WT or MORC2 N39A HCMV (MOI = 1). WT, parental WT HCMV; M, MORC2 WT HCMV; N, MORC2 N39A HCMV. Cells were harvested at the indicated time points. GAPDH served as a loading control (n = 2).
(TIF)

S5 Fig Generation and characterization of V5-tagged UL112-113 HCMV. (A) RFLP analysis of WT HCMV BAC and V5-UL112-113 HCMV BAC. BAC DNA was digested with XhoI and resolved by agarose gel electrophoresis to assess genome integrity and correct recombination. The red asterisk indicates the expected restriction fragment resulting from insertion of the V5-6xGGS linker. (B) Immunoblot analysis of viral proteins (IE1/2, UL44, and pp28) and V5-tagged UL112-113 in HFFs infected with WT or V5-UL112-113 HCMV (MOI = 1). Cells were harvested at the indicated time points. Vinculin served as a loading control. The black asterisks indicate V5-tagged UL112-113 isoforms. (C) Top, infectious progeny production was quantified by IFA of IE1/2-positive cells using cell-free supernatants collected at the indicated time points from HFFs infected with WT or V5-UL112-113 HCMV (MOI = 0.1) (mean ± SEM, n = 3). Bottom, representative immunofluorescence images of fresh HFFs infected with 10-fold diluted cell-free supernatants collected at 9 dpi. Cells were stained for IE1/2 (green) and counterstained with DAPI (blue). Two-way ANOVA with Sidak’s multiple comparisons test was performed across the time course; statistical comparisons at 7 and 9 dpi are shown. Scale bar, 200 μm. (D) Immunofluorescence analysis of HFFs infected with V5-UL112-113 HCMV (MOI = 1) and fixed at the indicated time points. UL44 (green), V5-UL112-113 (orange), MORC2 (violet), and DAPI (light blue) are shown. Scale bar, 5 μm.
(TIF)

S6 Fig Analysis of the MORC2 IDR and generation of MORC2 ΔCC3 HCMV. (A) Prediction of intrinsically disordered regions in MORC2 using IUPred3. Using a disorder score threshold of 0.5, the extended region spanning amino acids 523-799 was selected as the predicted IDR for subsequent deletion analysis. (B) Schematic representation of the MORC2 ΔIDR mutant. The predicted IDR spanning amino acids 523-799 was deleted, and an SV40 NLS flanked on both sides by 2xGGGGS linkers was introduced to preserve nuclear localization. (C) RFLP analysis of WT HCMV, MORC2 WT HCMV, and MORC2 ΔCC3 HCMV BACs. BAC DNA was digested with BamHI and resolved by agarose gel electrophoresis. The red asterisks indicate the expected restriction fragment changes resulting from MORC2 insertion and subsequent CC3 deletion in MORC2 WT and MORC2 ΔCC3 HCMV BACs, respectively.
(TIF)

S1 Table Primers used for quantitative PCR. (XLSX)

S2 Table Homology arm sequences used for BAC recombination. (XLSX)

S1 Raw Gel Original images for blots and gels. (PDF)
