# Supplementary material for: Human cytomegalovirus exploits host chromatin remodeler MORC2 to promote the DNA damage response on viral genomes
Source: PLoS Pathog. 2026 Oct 14;22(10):e1014693. doi: 10.1371/journal.ppat.1014693 (PMC13693984; doi:10.1371/journal.ppat.1014693)

Original images for blots and gels

Figure 1

Fig 1A

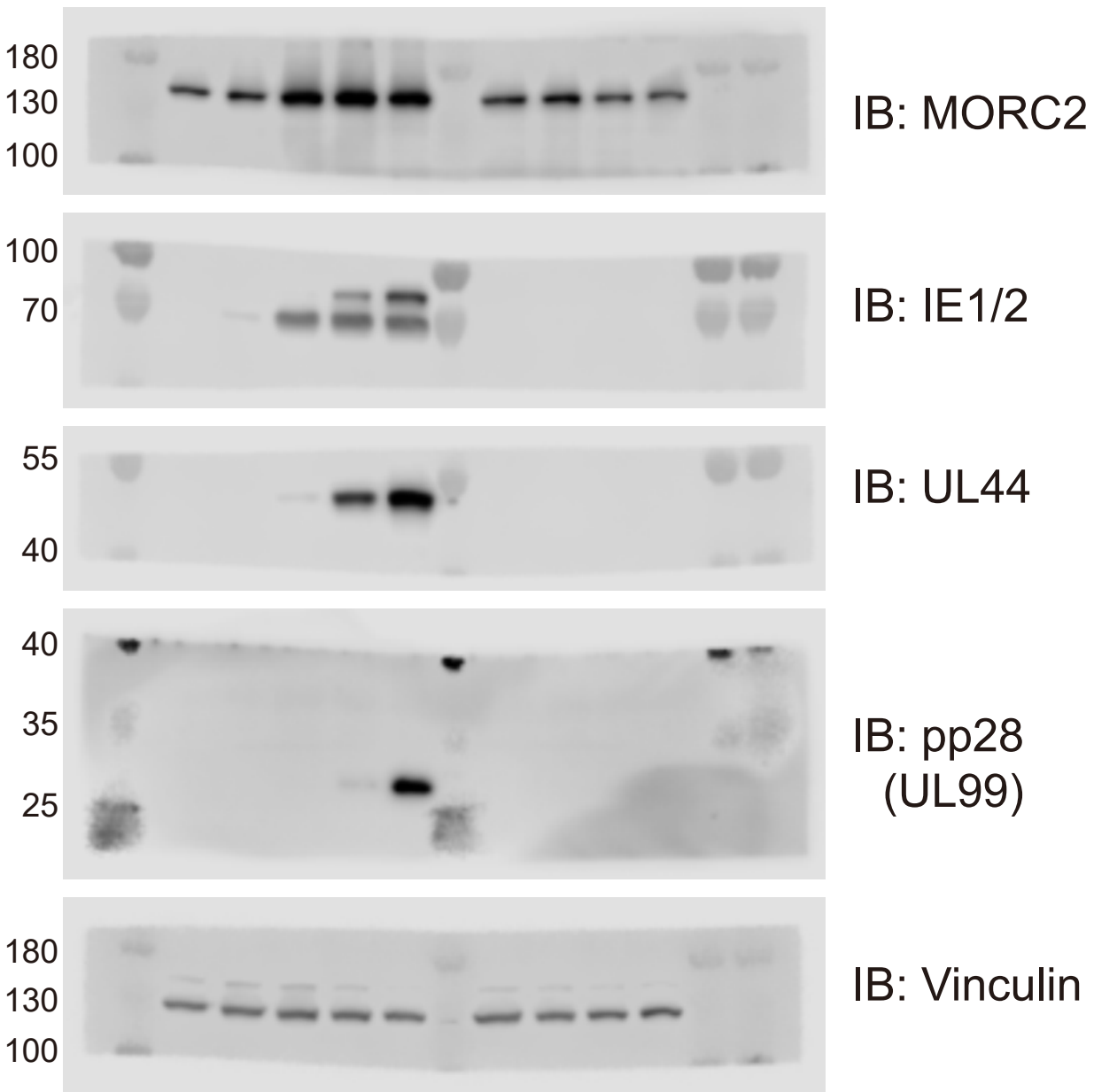

Fig 1D

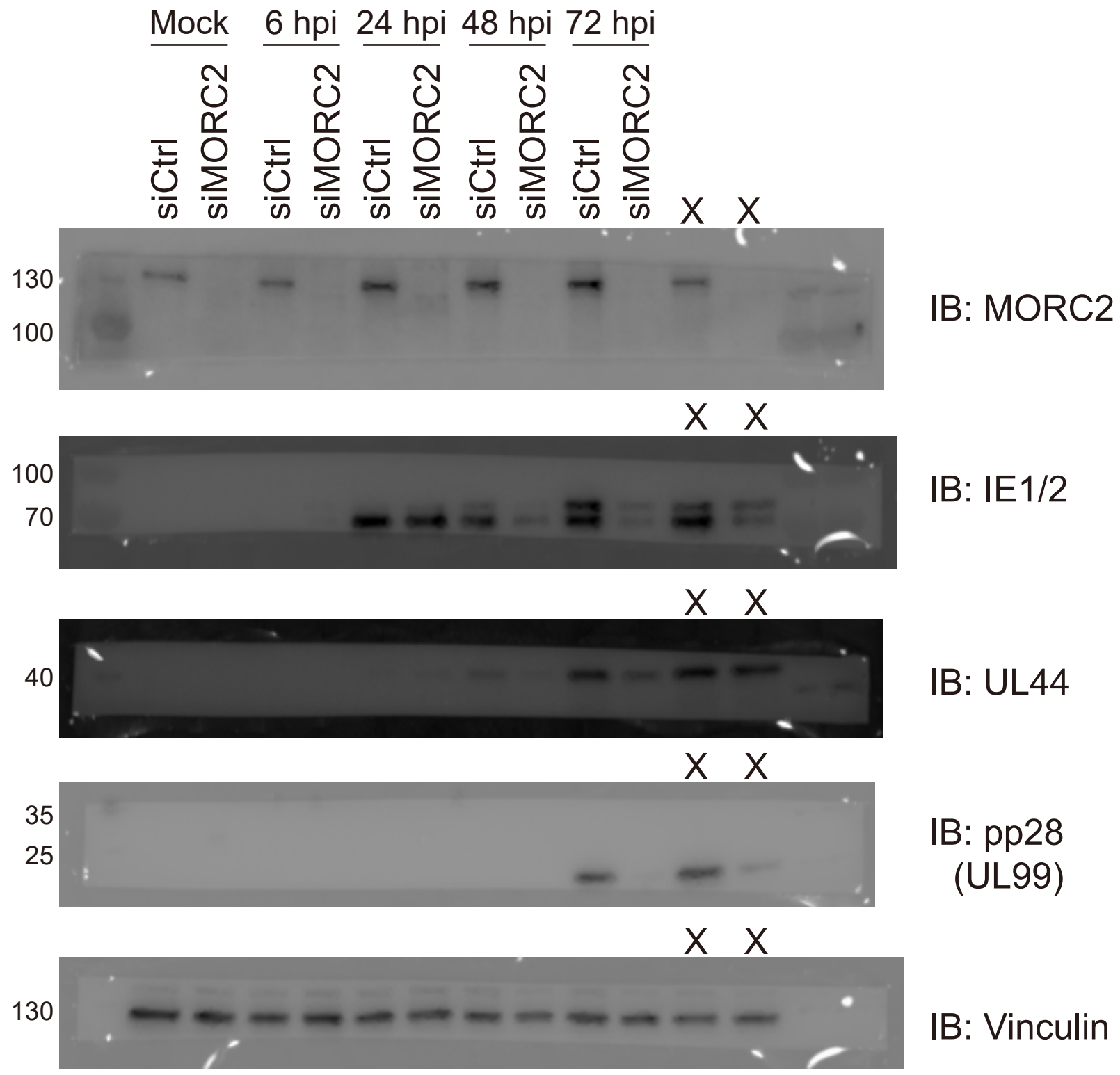

Fig 1J

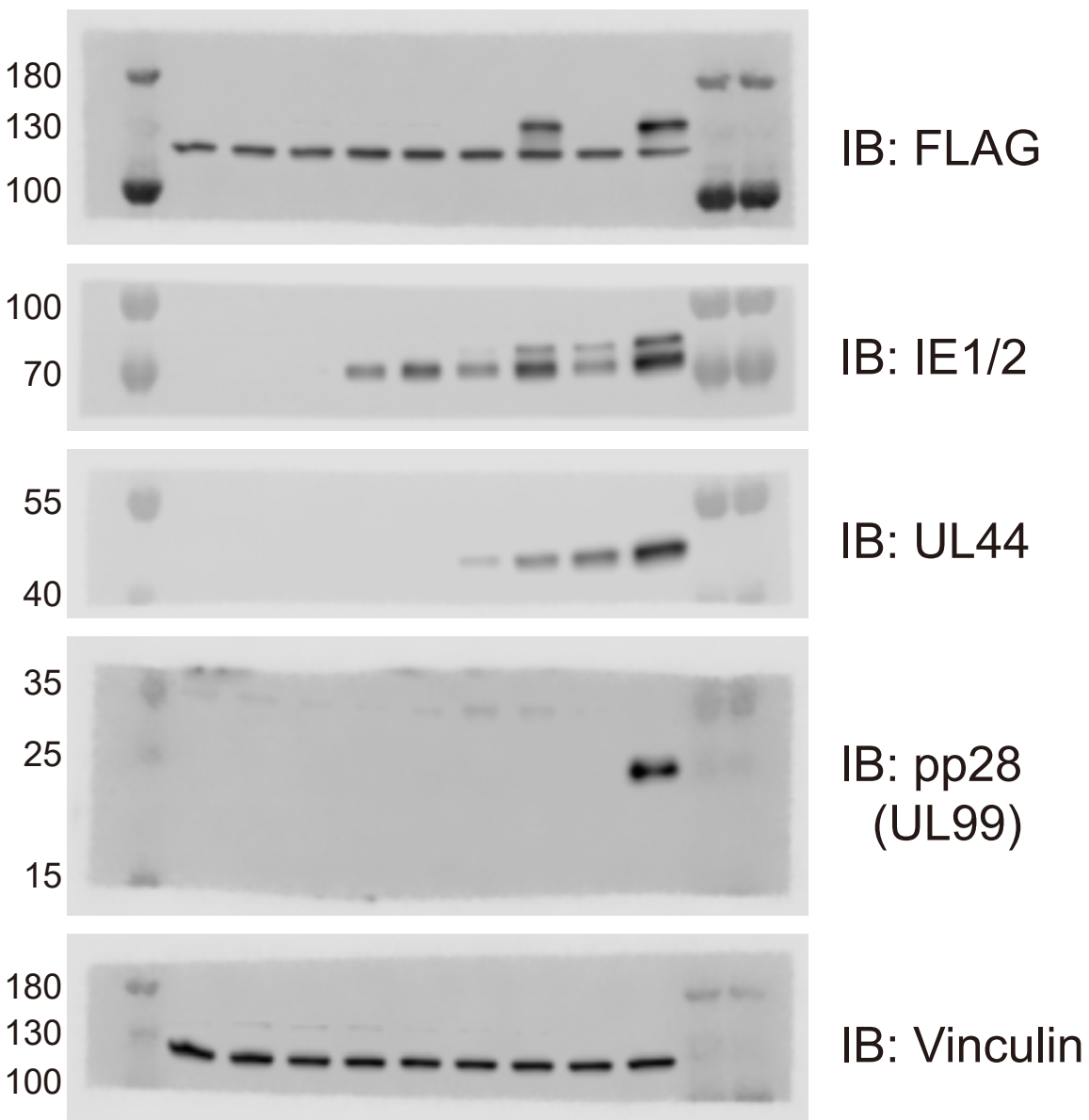

Image acquisition: Immunoblot images were acquired by chemiluminescence using an Odyssey XF Imaging System (LI-COR Biosciences), except for Fig 1D, S1C, S2A, S3A, and S3B, which were acquired using a Fusion SL2-3500.WL imaging system (Vilber Lourmat). Agarose gel images were captured using a smartphone camera.

Original images for blots and gels

Figure 4

Fig 4D

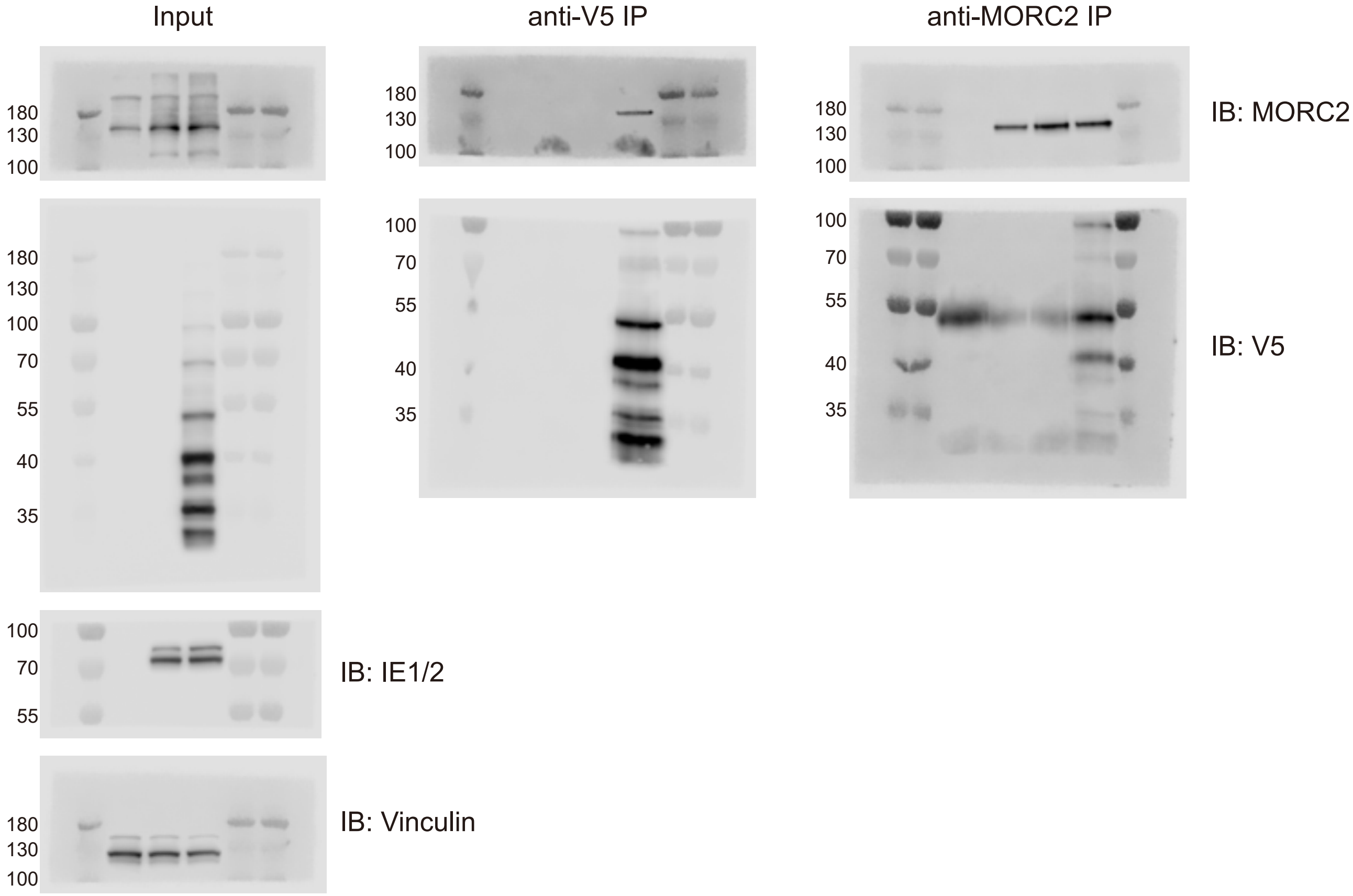

Fig 4F

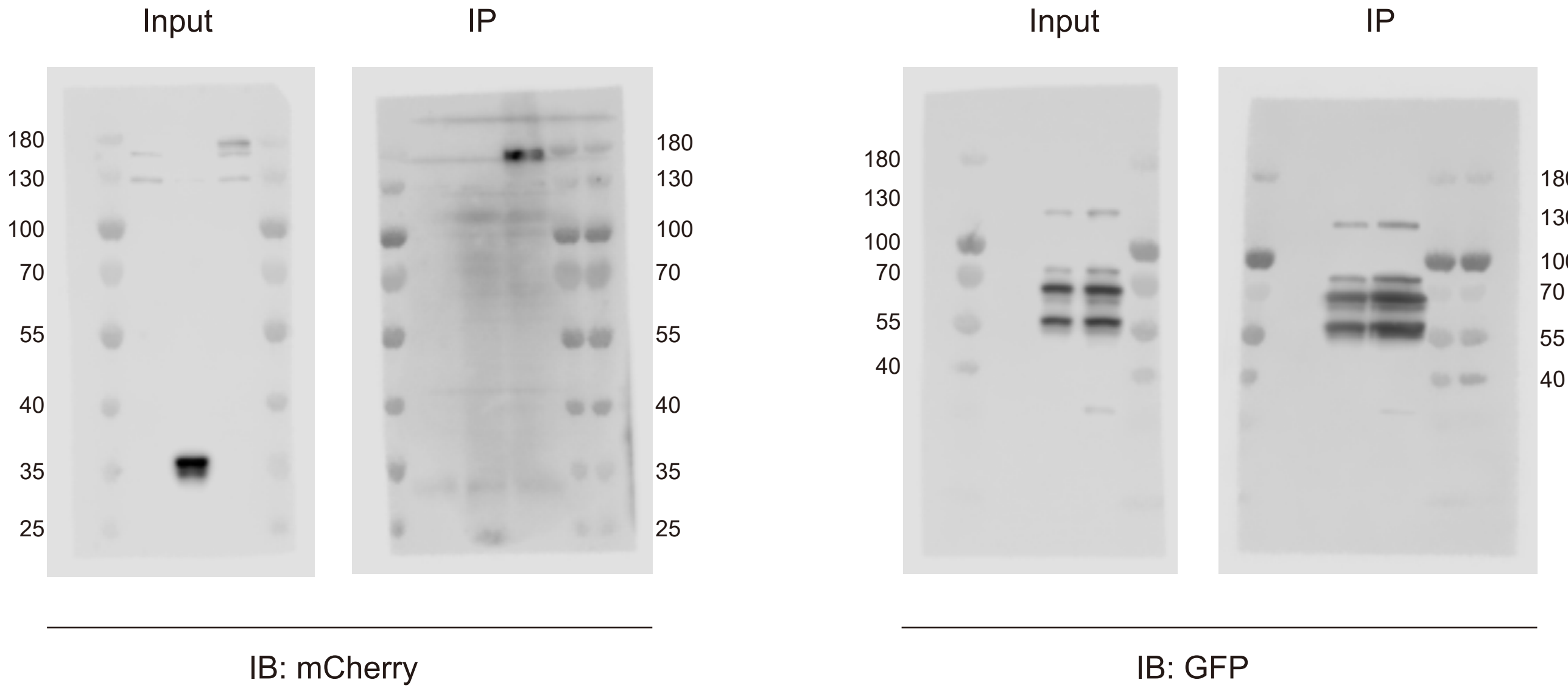

Original images for blots and gels

Figure 5

Fig 5D

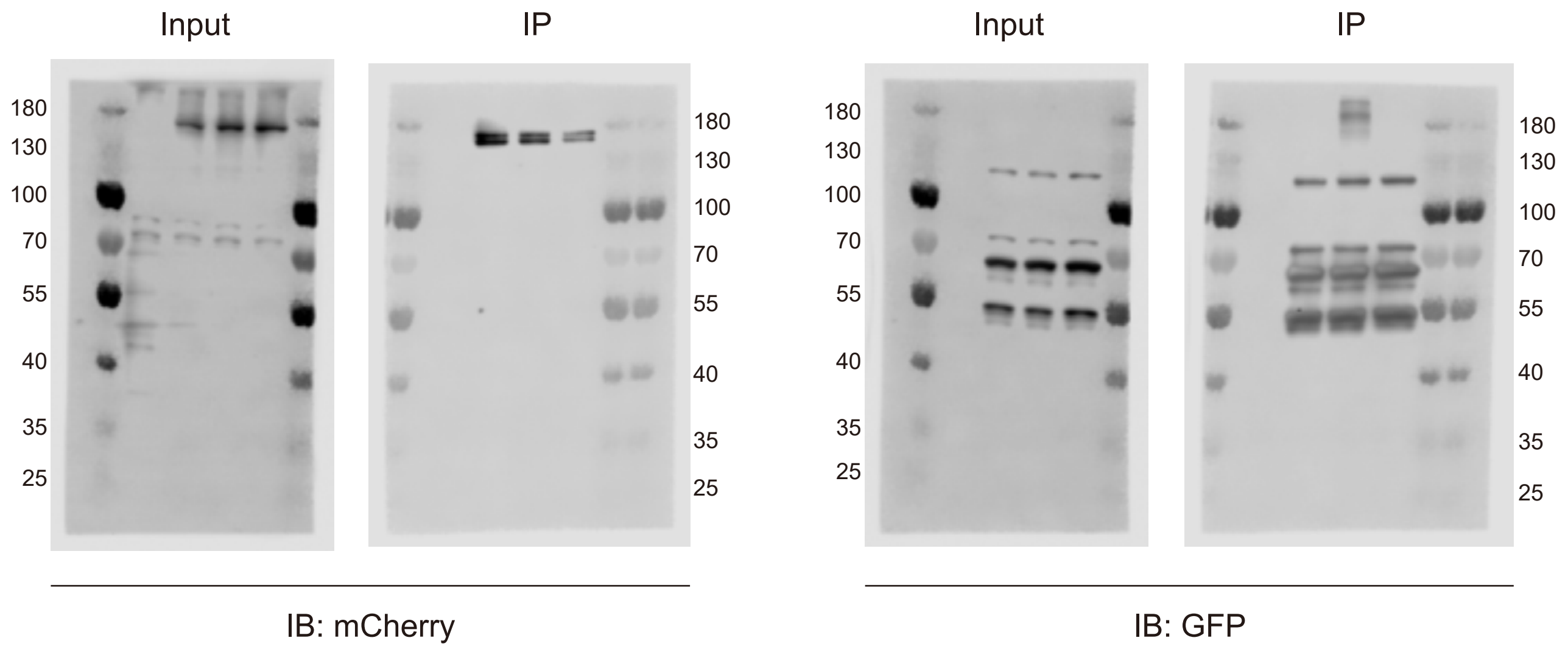

Figure 6

Fig 6C

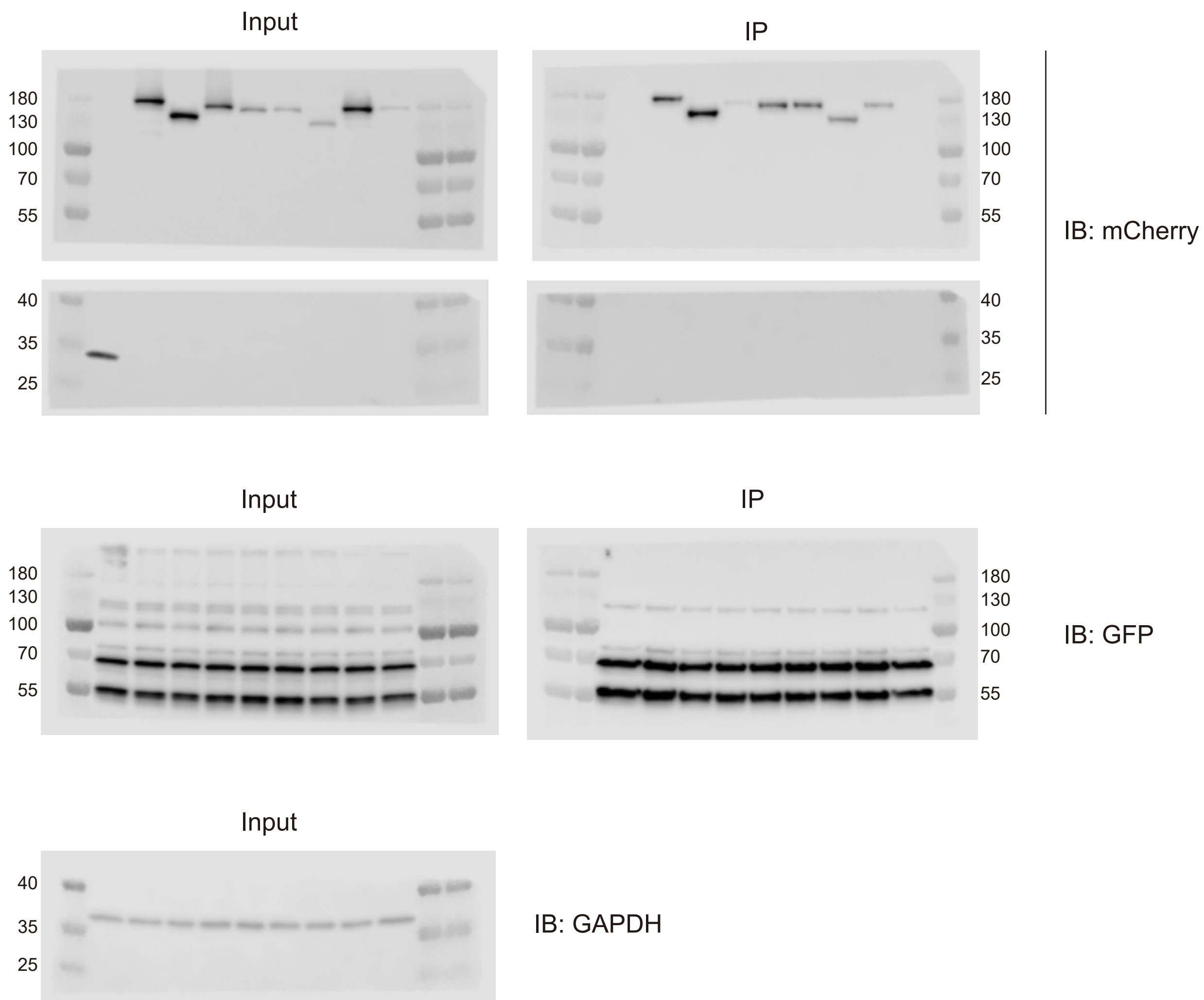

Original images for blots and gels

Supplementary Figure 1

Fig S1A

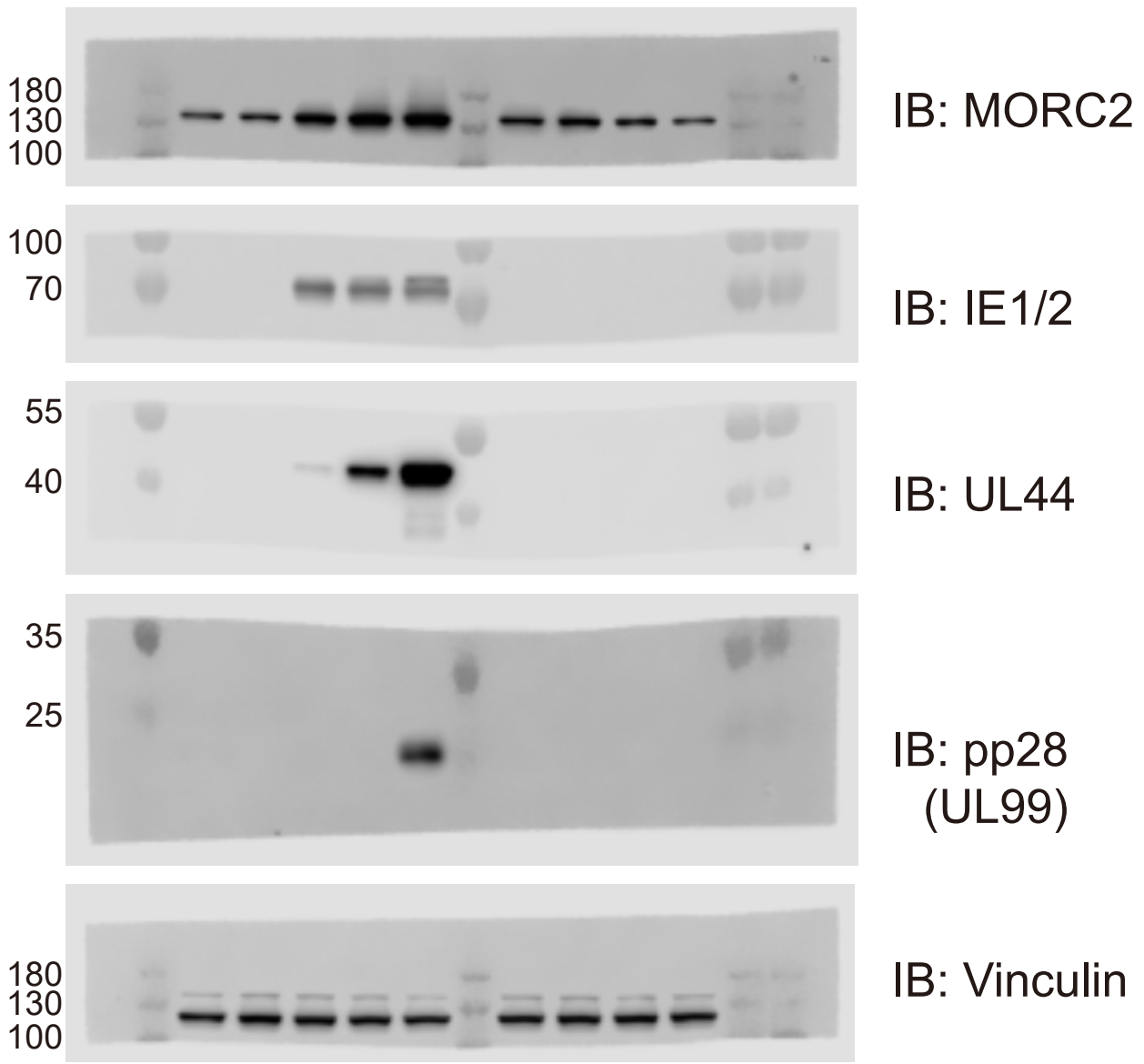

Fig S1C

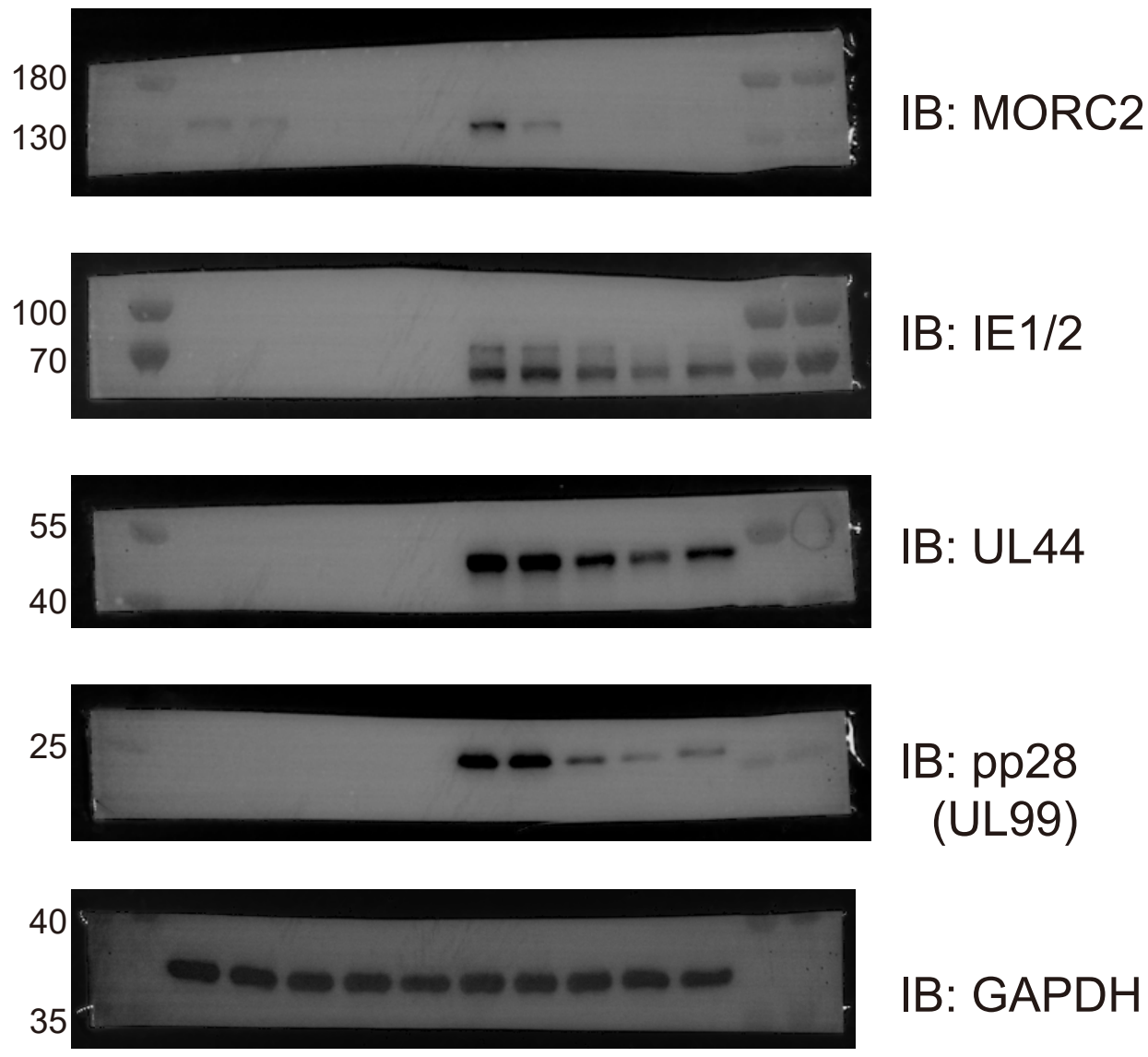

Fig S1D

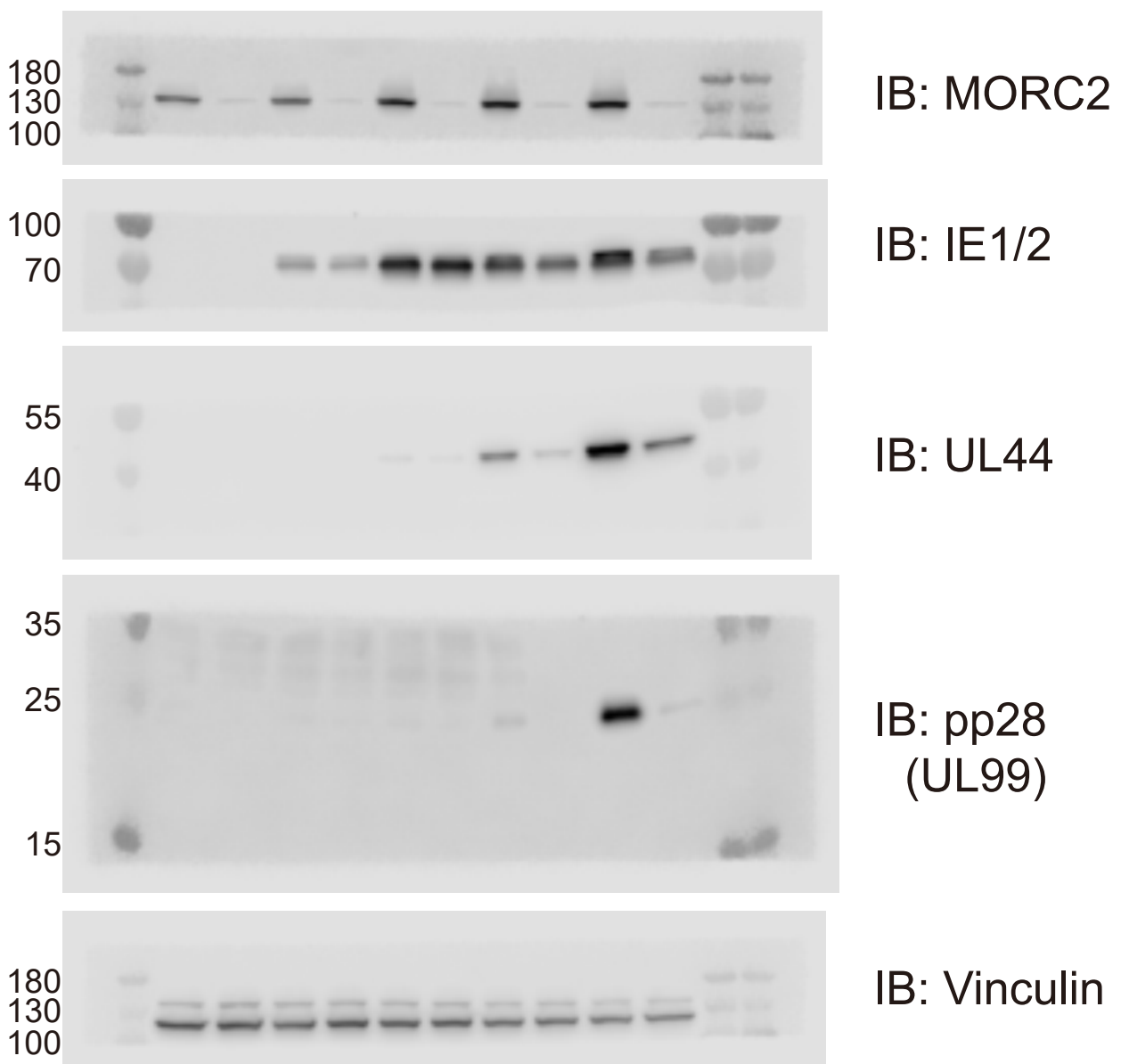

Fig S1F

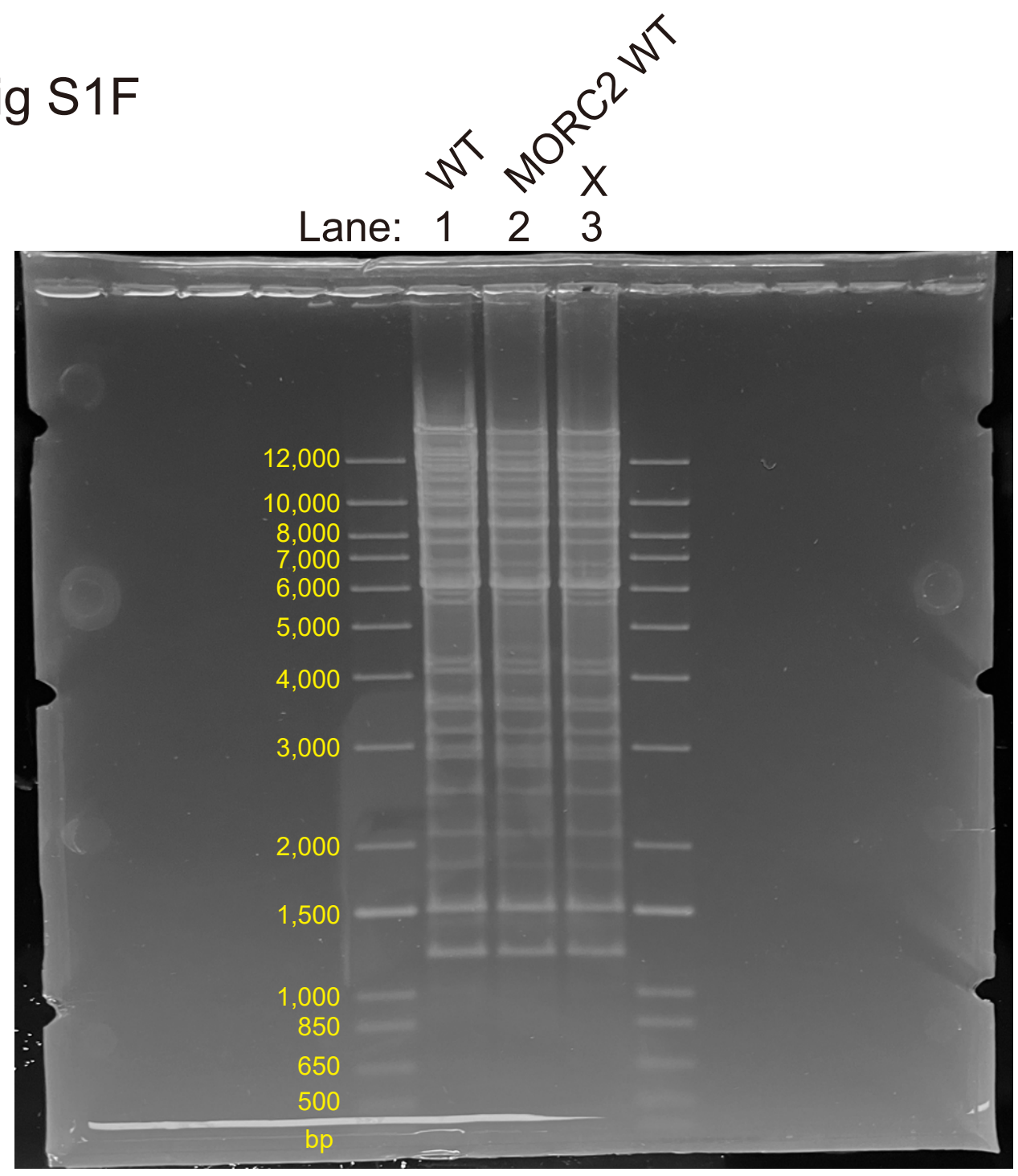

Original images for blots and gels

Supplementary Figure 2

Fig S2A

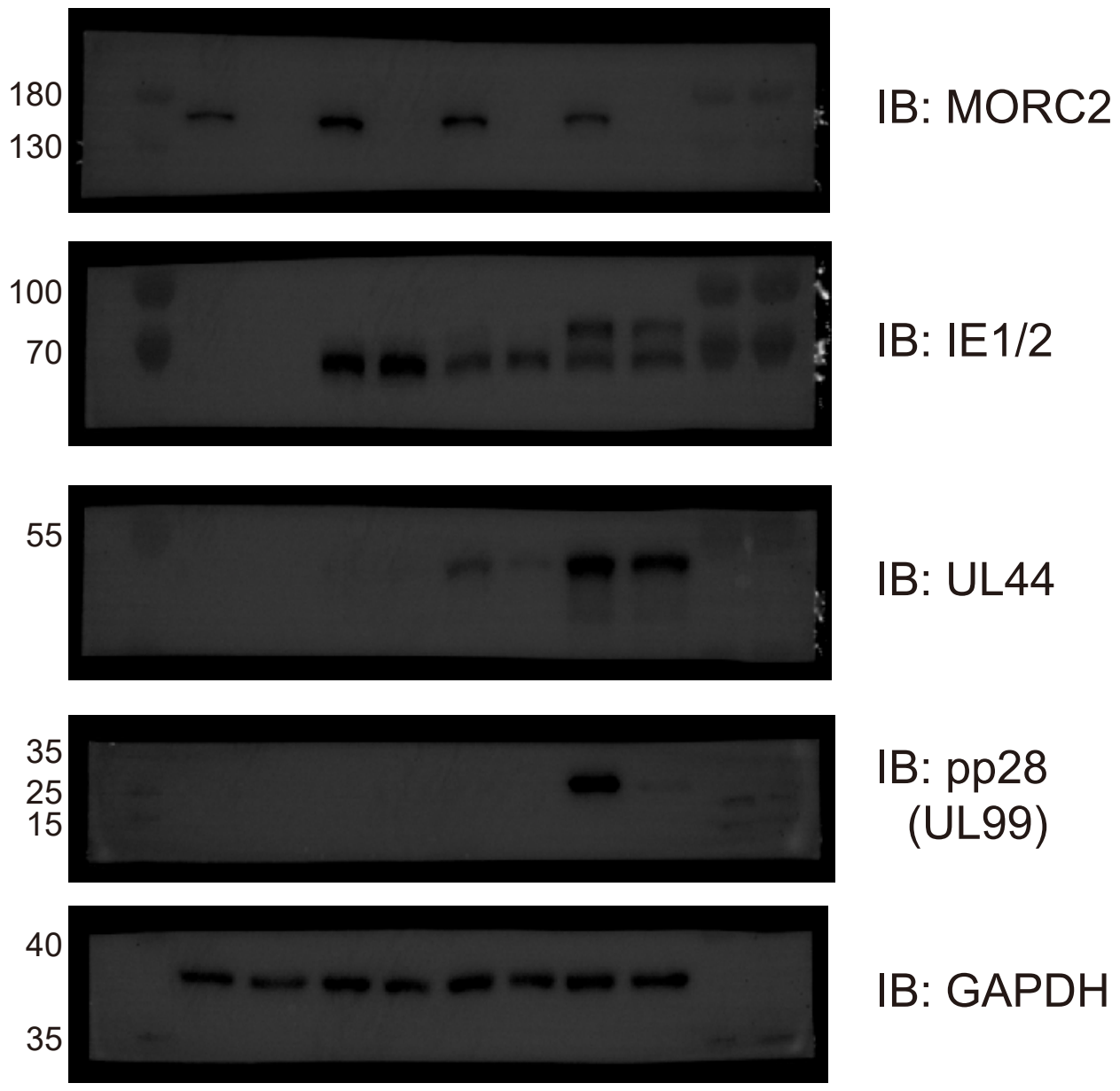

Fig S2B

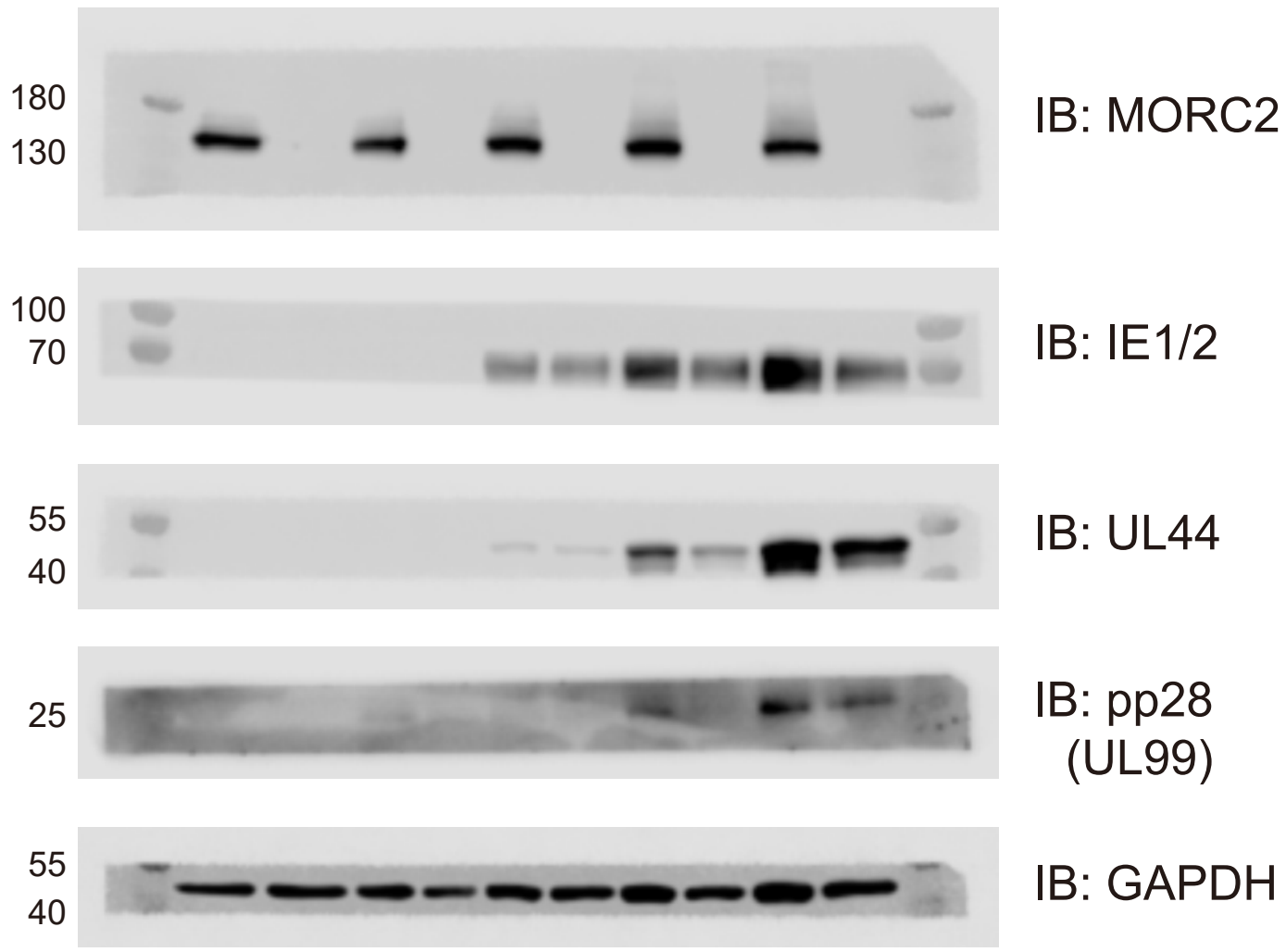

Original images for blots and gels

Supplementary Figure 3

Fig S3A

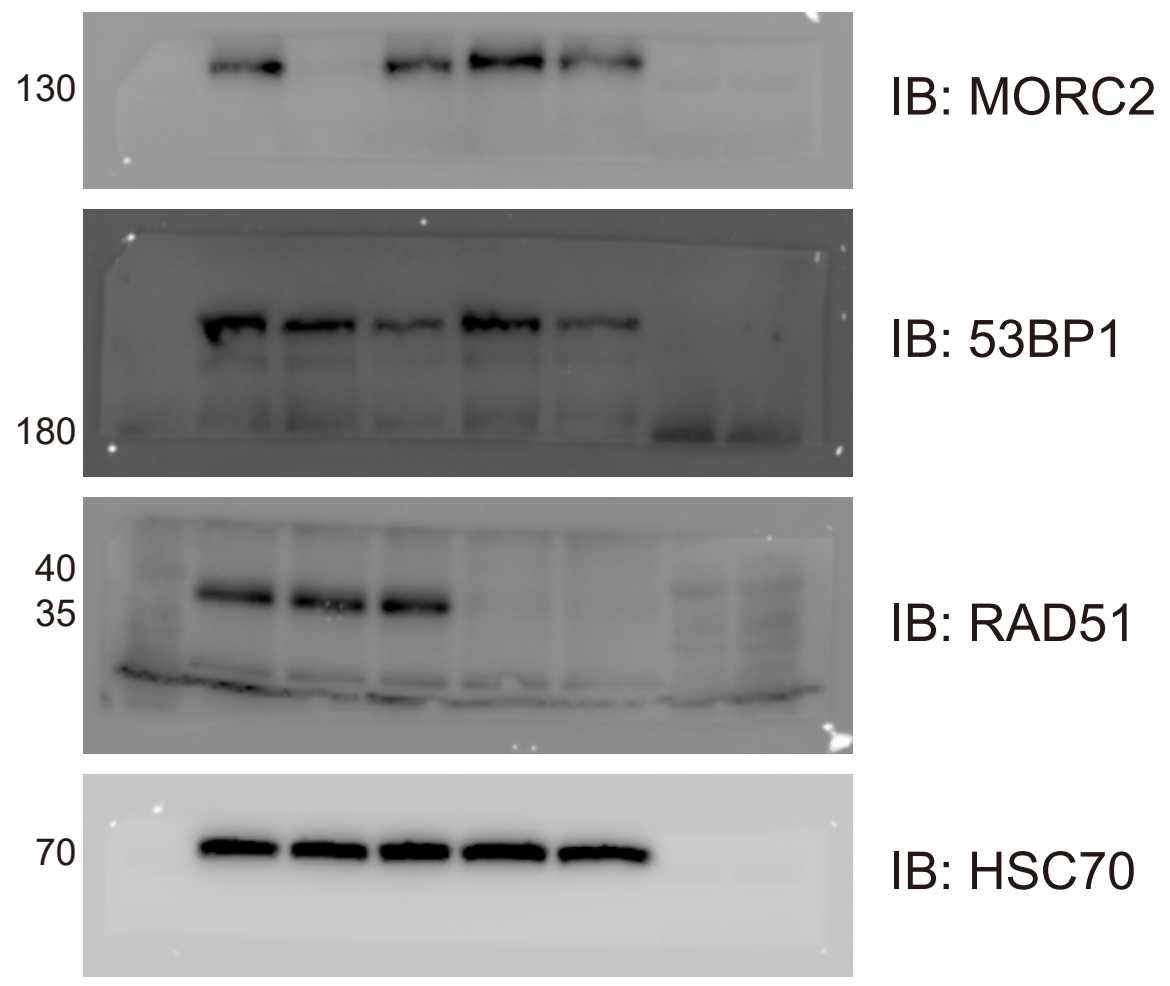

Fig S3B

Mock, 24 hpi

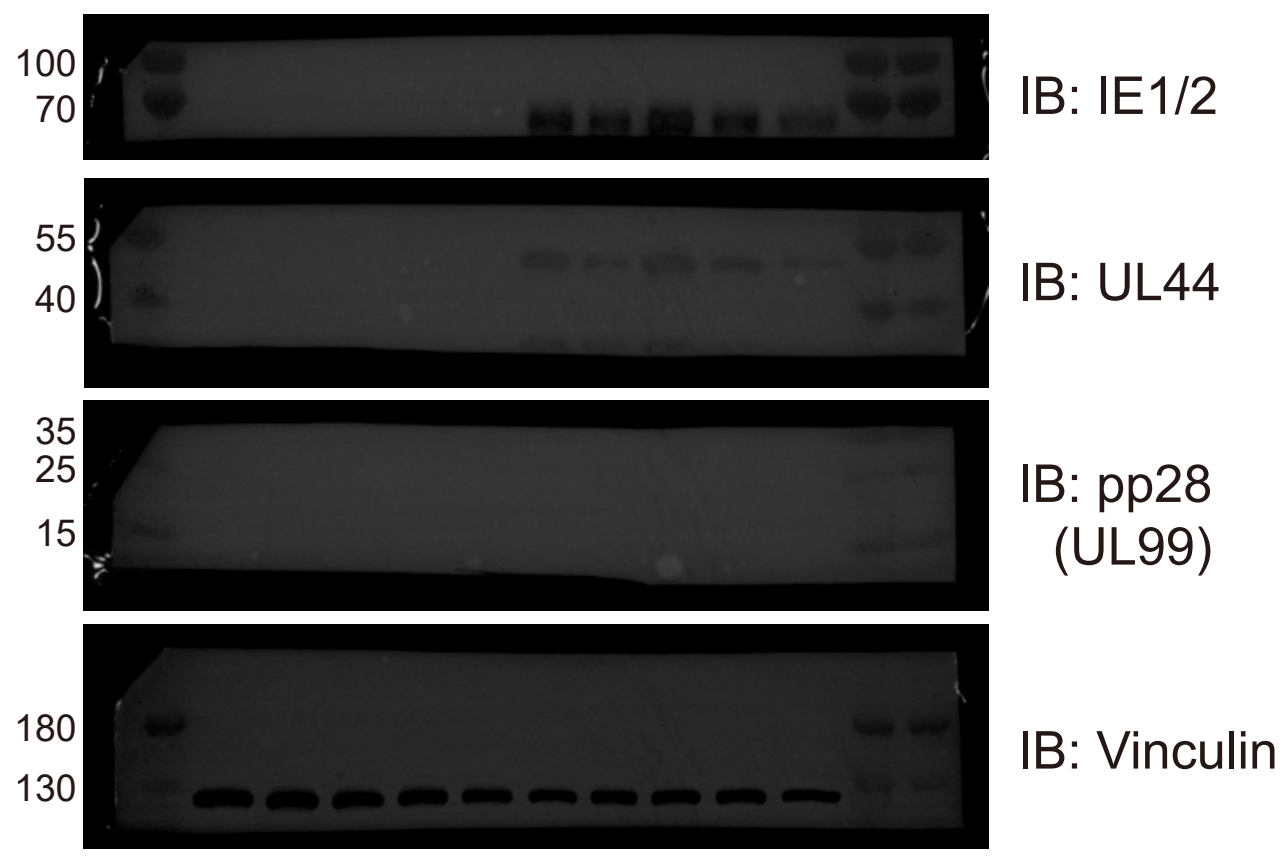

48, 72 hpi

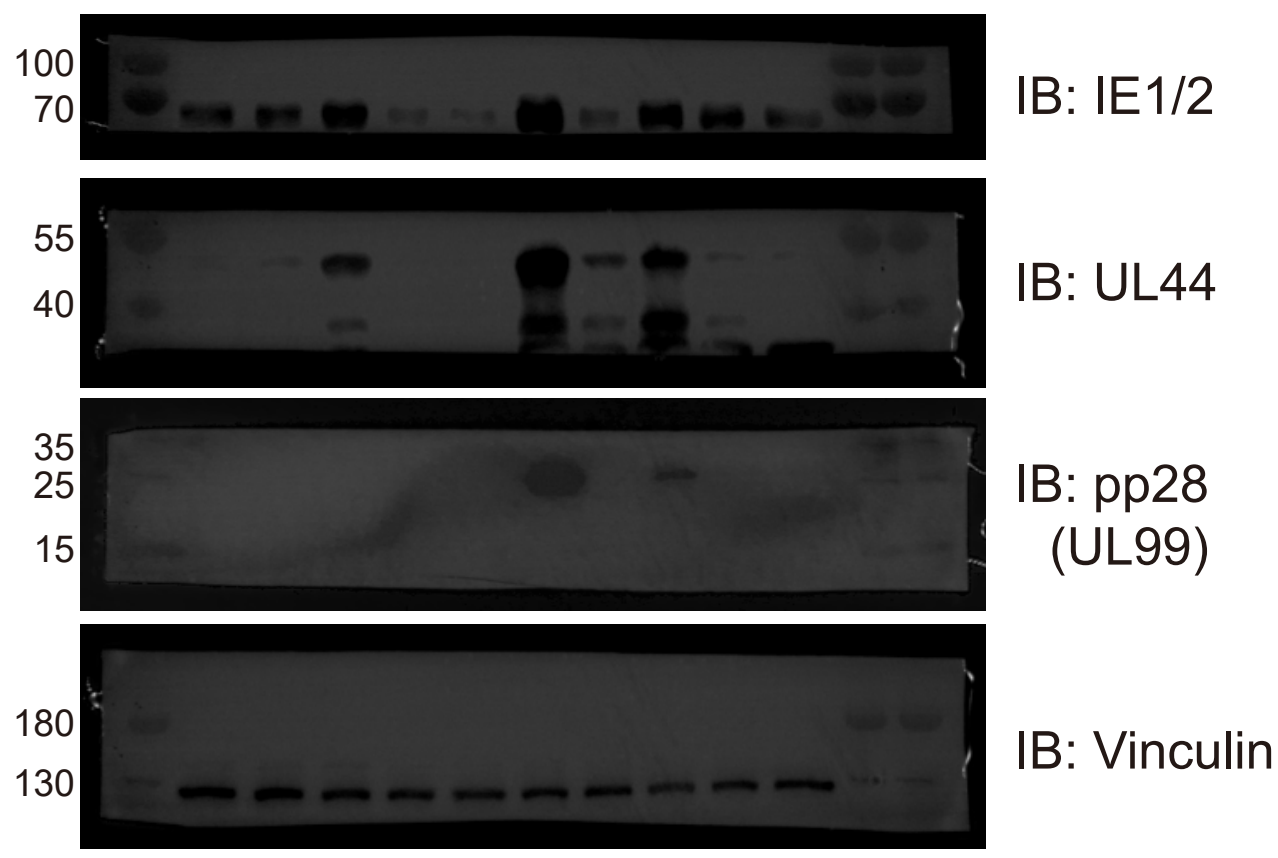

Original images for blots and gels

Supplementary Figure 4

Fig S4A

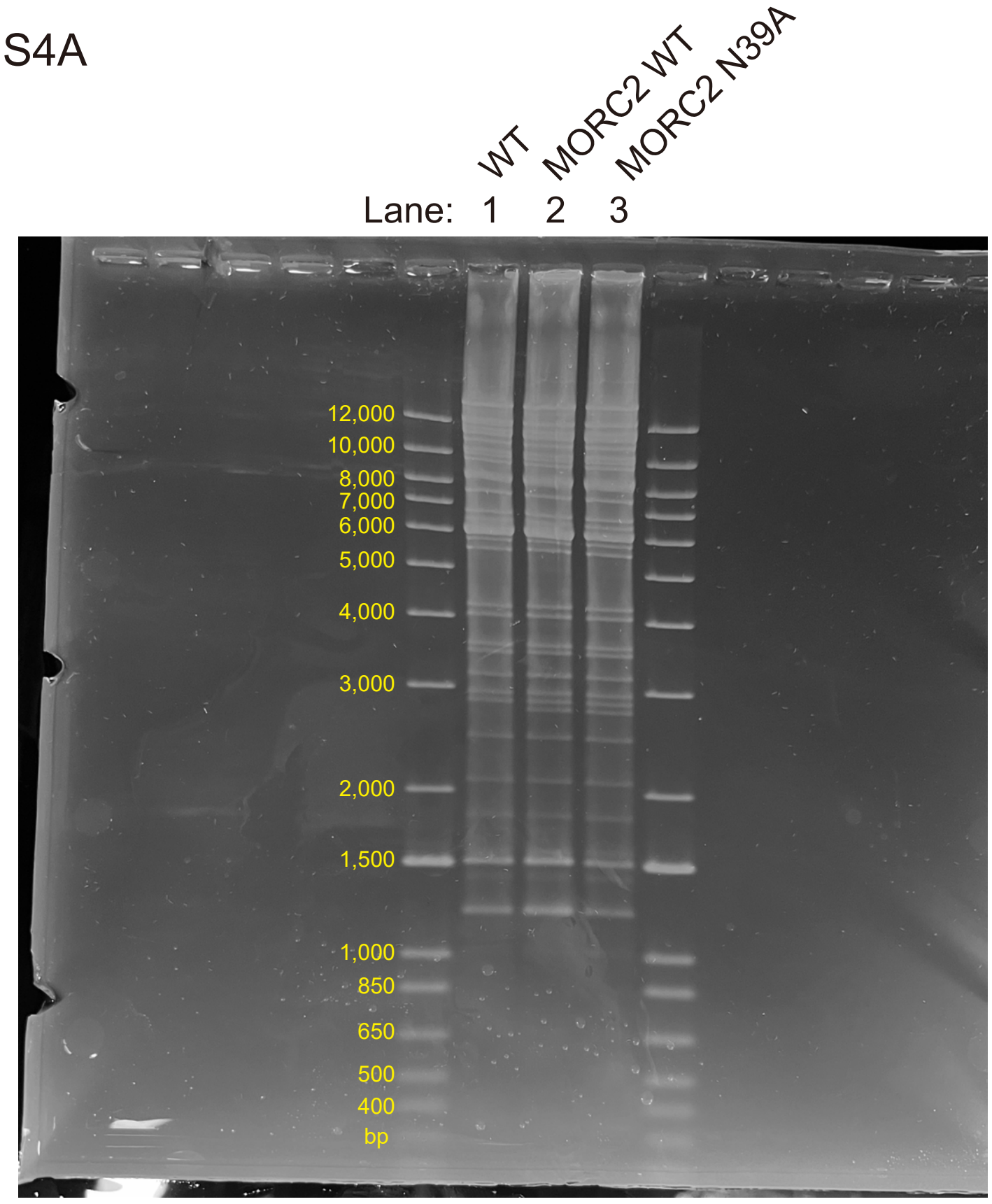

Fig S4C

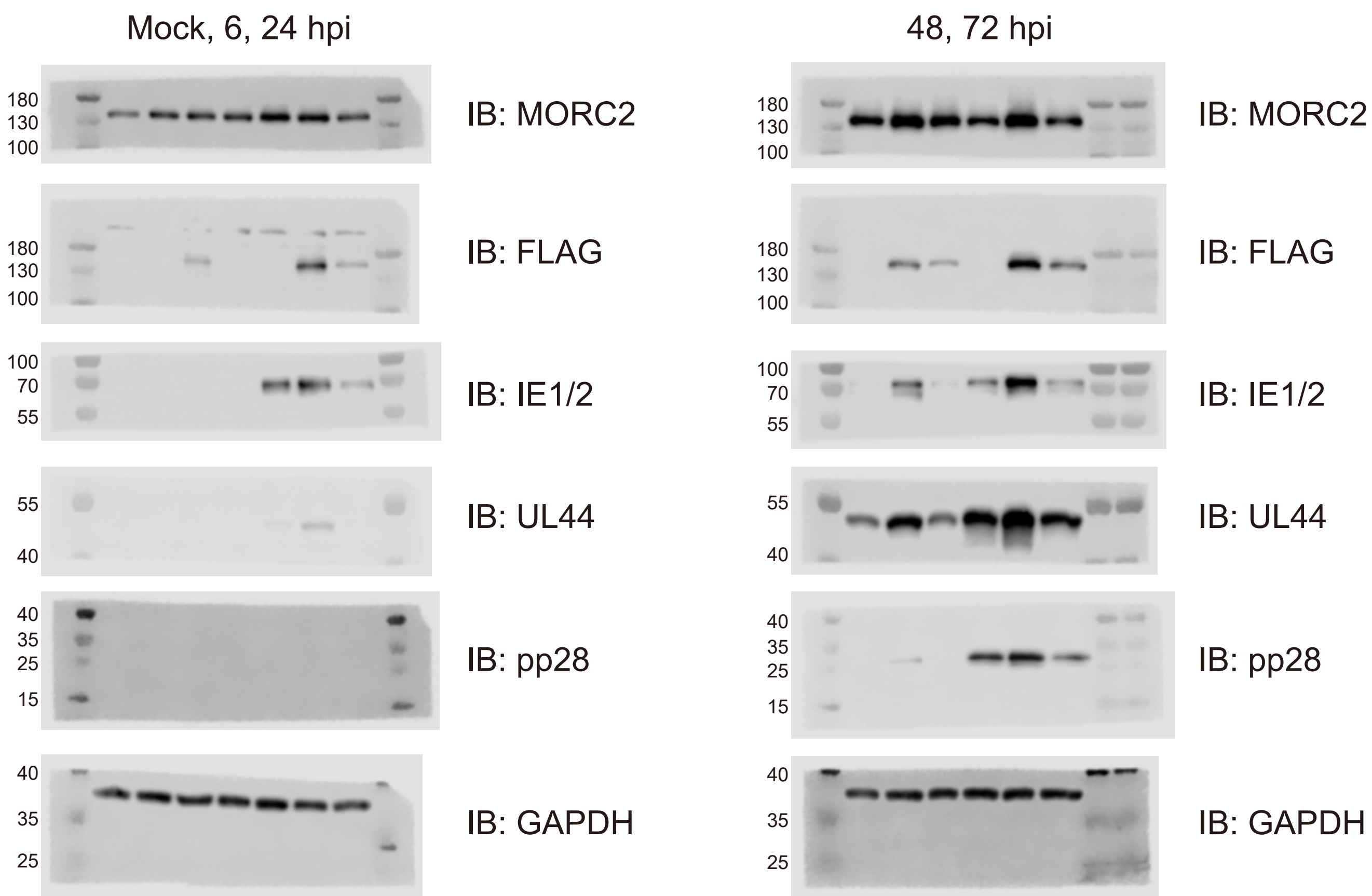

Original images for blots and gels

Supplementary Figure 5

Fig S5A

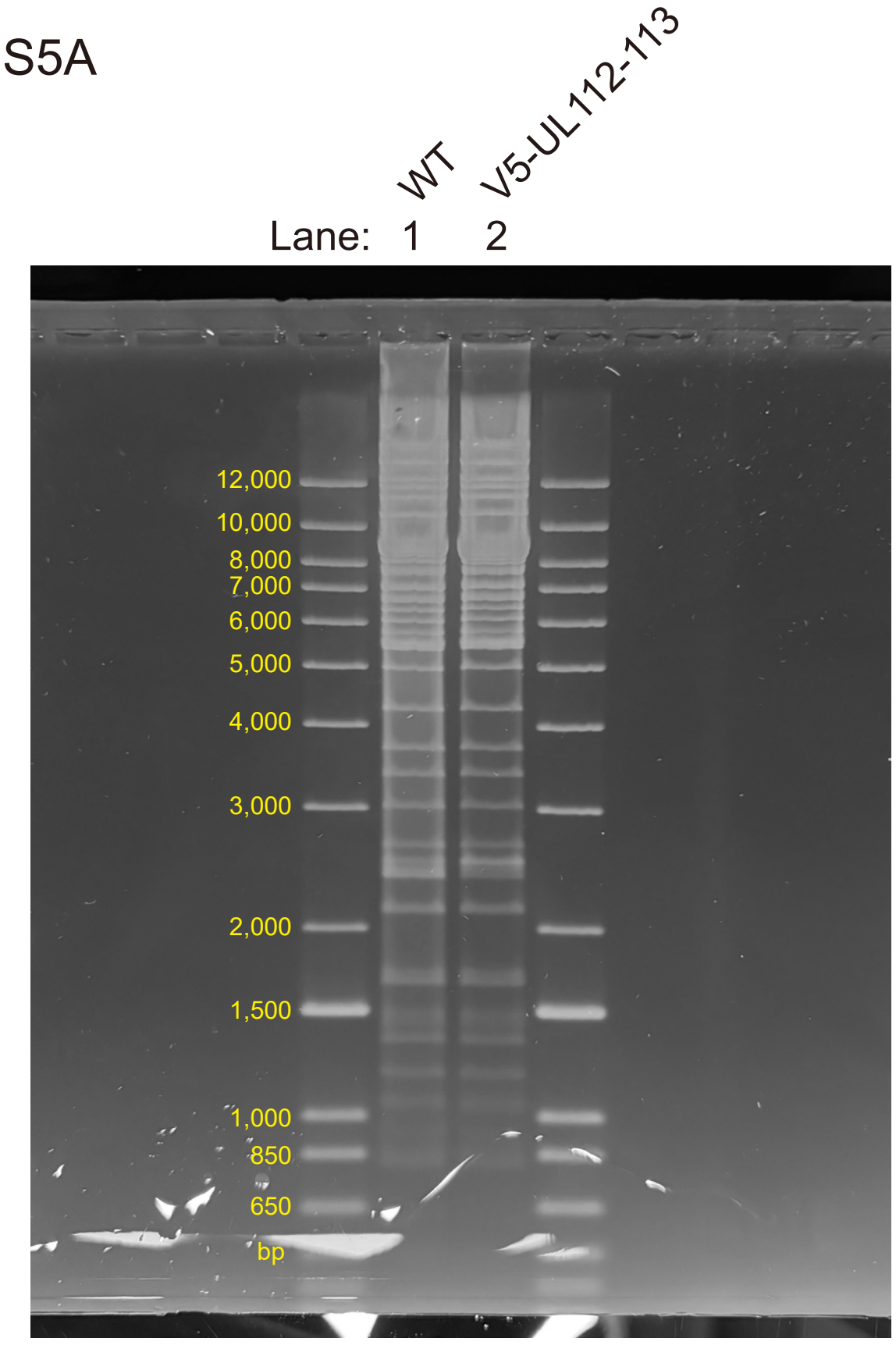

Fig S5B

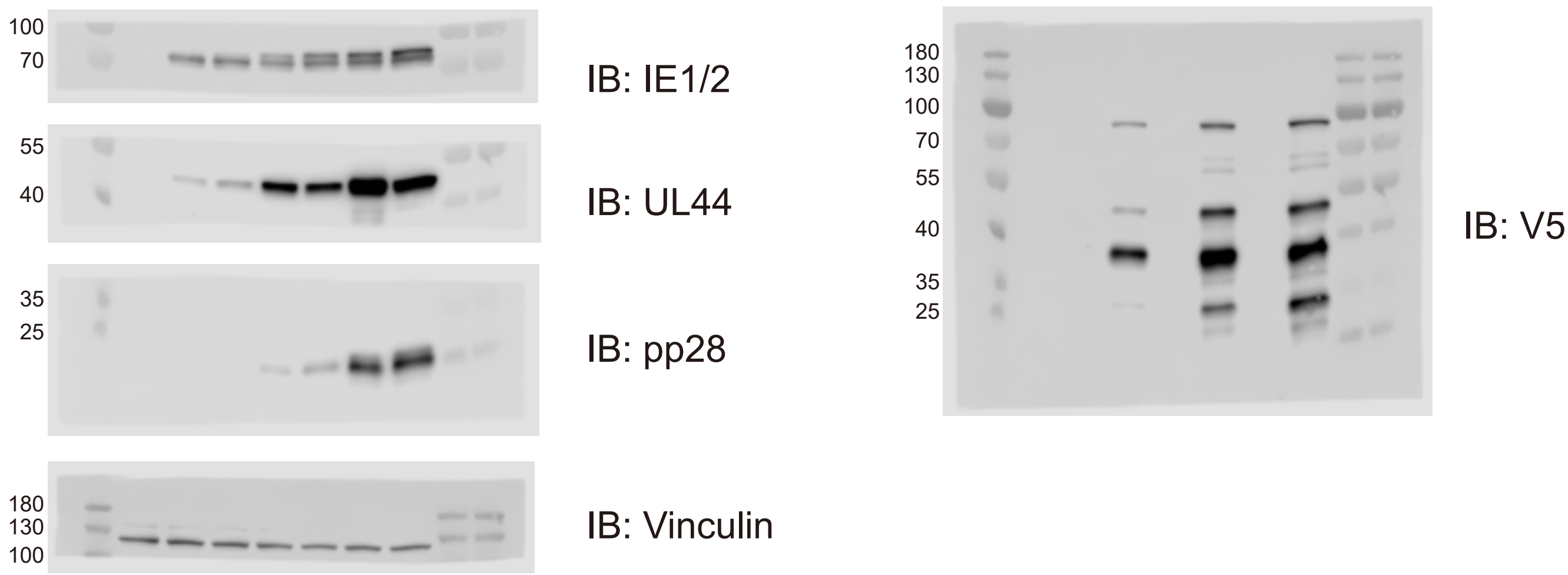

Original images for blots and gels

Supplementary Figure 6

Fig S6C

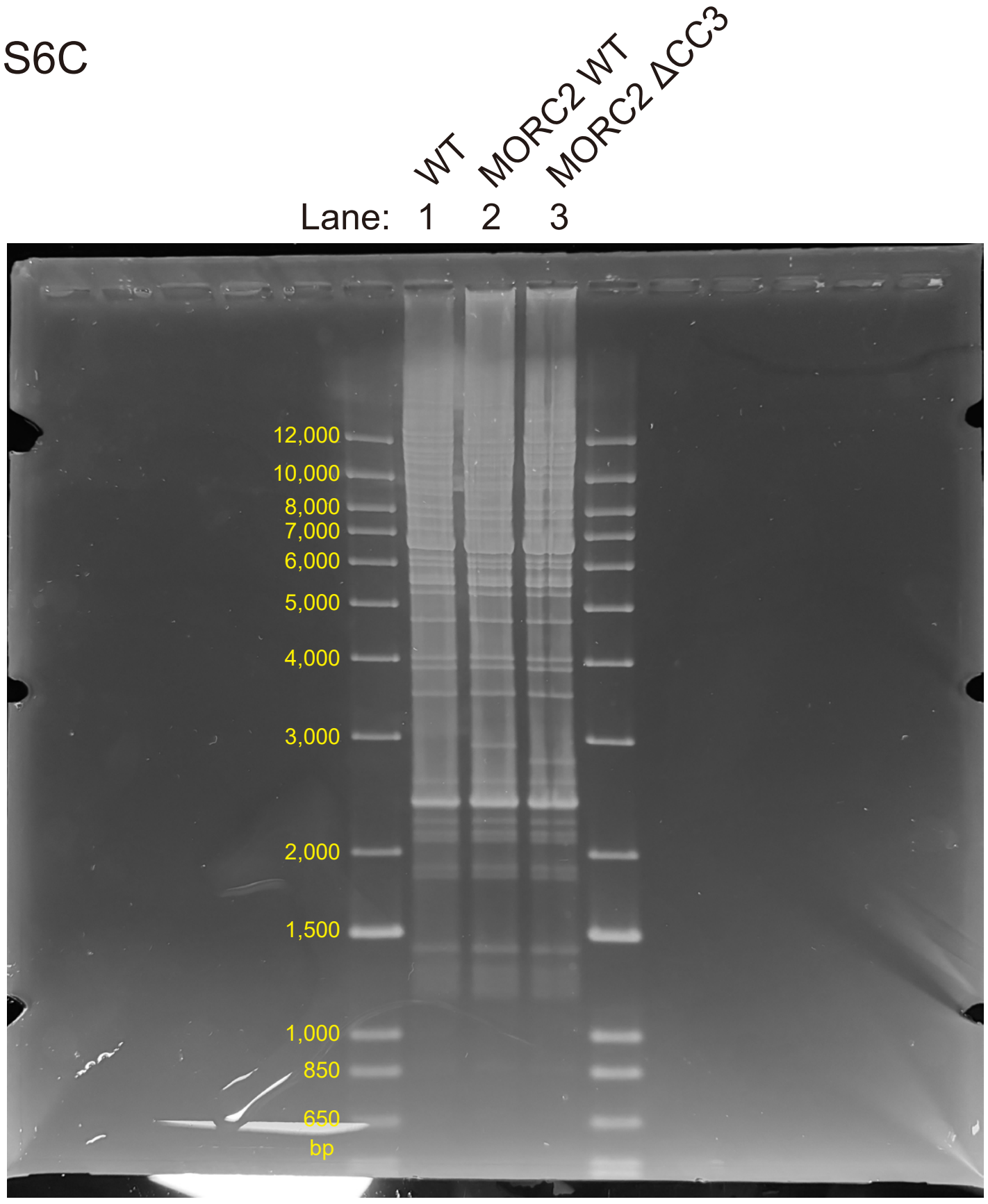

Supplement: Supplementary file 1 [file ppat.1014693.s009.pdf]
